# Age-related Changes in Lateral Entorhinal and CA3 Neuron Allocation Predict Poor Performance on Object Discrimination

**DOI:** 10.3389/fnsys.2017.00049

**Published:** 2017-06-30

**Authors:** Andrew P. Maurer, Sarah A. Johnson, Abbi R. Hernandez, Jordan Reasor, Daniela M. Cossio, Kaeli E. Fertal, Jack M. Mizell, Katelyn N. Lubke, Benjamin J. Clark, Sara N. Burke

**Affiliations:** ^1^Department of Neuroscience, McKnight Brain Institute, University of FloridaGainesville, FL, United States; ^2^Department of Biomedical Engineering, University of FloridaGainesville, FL, United States; ^3^UF Summer Neuroscience Internship Program, Department of Neuroscience, McKnight Brain Institute, University of FloridaGainesville, FL, United States; ^4^Department of Psychology, University of New MexicoAlburquerque, NM, United States; ^5^Department of Aging and Geriatric Research, UF Institute on Aging, University of FloridaGainesville, FL, United States

**Keywords:** *Arc*, hippocampus, immediate-early gene, memory, retrograde tracer

## Abstract

Age-related memory deficits correlate with dysfunction in the CA3 subregion of the hippocampus, which includes both hyperactivity and overly rigid activity patterns. While changes in intrinsic membrane currents and interneuron alterations are involved in this process, it is not known whether alterations in afferent input to CA3 also contribute. Neurons in layer II of the lateral entorhinal cortex (LEC) project directly to CA3 through the perforant path, but no data are available regarding the effects of advanced age on LEC activity and whether these activity patterns update in response to environmental change. Furthermore, it is not known the extent to which age-related deficits in sensory discrimination relate to the inability of aged CA3 neurons to update in response to new environments. Young and aged rats were pre-characterized on a LEGO^©^ object discrimination task, comparable to behavioral tests in humans in which CA3 hyperactivity has been linked to impairments. The cellular compartment analysis of temporal activity with fluorescence *in situ* hybridization for the immediate-early gene Arc was then used to identify the principal cell populations that were active during two distinct epochs of random foraging in different environments. This approach enabled the extent to which rats could discriminate two similar objects to be related to the ability of CA3 neurons to update across different environments. In both young and aged rats, there were animals that performed poorly on the LEGO object discrimination task. In the aged rats only, however, the poor performers had a higher percent of CA3 neurons that were active during random foraging in a novel environment, but this is not related to the ability of CA3 neurons to remap when the environment changed. Afferent neurons to CA3 in LEC, as identified with the retrograde tracer choleratoxin B (CTB), also showed a higher percentage of cells that were positive for Arc mRNA in aged poor performing rats. This suggests that LEC contributes to the hyperactivity seen in CA3 of aged animals with object discrimination deficits and age-related cognitive decline may be the consequence of dysfunction endemic to the larger network.

## Introduction

The hippocampal circuit is vulnerable to normative aging processes, which has been linked to age-related memory loss (Rosenzweig and Barnes, 2003; Wilson et al., 2005, 2006; Burke and Barnes, 2006, 2010). While all subregions of hippocampus show age-related alterations, recently hyperactivity in CA3 has been reported in humans (Yassa et al., 2010a, 2011), monkeys (Thome et al., 2015), and rats (Wilson et al., 2005; Robitsek et al., 2015). There is also a cross-species consensus that the perforant path from the entorhinal cortex to the hippocampus losses fibers in old age (Barnes and McNaughton, 1980; Foster et al., 1991; Yassa et al., 2010b, 2011), despite intact cell numbers in the entorhinal cortex (Rapp et al., 2002). Reduced perforant path input could attenuate feedforward inhibition to CA3 (Buzsaki and Czeh, 1981; Buzsaki, 1984). Thus, together with interneuron dysfunction (Spiegel et al., 2013) and enhanced A-type K^+^ currents (Simkin et al., 2015), this fiber loss could promote CA3 hyperexcitability (Wilson et al., 2005; Yassa et al., 2010a, 2011; Robitsek et al., 2015). In fact, the degree of perforant pathway loss correlates with levels of CA3 hyperexcitability in humans (Yassa et al., 2011). Importantly, the lateral portion of the origin of the perforant path in the entorhinal cortex is among the earliest sites of dysfunction in Alzheimer's disease with neurofibrillary tangles forming in that region prior to hippocampus and other cortical areas (Braak et al., 1993, 2006). Early vulnerability within this region has been recapitulated in animal models of Alzheimer's disease (Khan et al., 2014), however, little is known regarding the integrity of the lateral entorhinal cortex (LEC) in normal aging.

Despite accumulating evidence that the lateral entorhinal cortical-perforant path to CA3 circuit is vulnerable in both normal aging and disease, interactions between these two brain areas have not yet been examined on the cellular level, and it is not known the extent to which lateral entorhinal cortical input contributes to CA3 hyperexcitability. It is critical to understand this interaction as lesion data have shown that the entorhinal input to CA3 is necessary for acquisition and retrieval of spatial memory (Lee and Kesner, 2004). Moreover, both cortical input into CA3 (Smith et al., 2000; Dieguez and Barea-Rodriguez, 2004) and spatial memory performance are known to degrade with advanced age (e.g., Barnes, 1979; Barnes et al., 1980; Buzsaki et al., 1986; Gallagher et al., 1993; Rosenzweig and Barnes, 2003; Bunzeck and Duzel, 2006). All of which could be related to rigid activity patterns of aged CA3 neurons that do not update across different environments (Wilson et al., 2005; Robitsek et al., 2015). Finally, traditional models contend that memory requires an ability to discriminate between similar stimuli and events, which theoretically relies on a balance between the competing processes of pattern separation and pattern completion within the dentate gyrus-CA3 network (McNaughton and Morris, 1987; Treves and Rolls, 1994; McClelland et al., 1995; O'Reilly and Norman, 2002; Norman and O'Reilly, 2003; Yassa and Stark, 2011; Rolls, 2013, 2015). This theoretical framework has led to a perspective in which CA3 hyperactivity in old animals impairs pattern separation in favor of pattern completion (Wilson et al., 2005, 2006; Yassa et al., 2010a, 2011; Bakker et al., 2012) manifesting as a behavioral impairment in the ability to discriminate between similar stimuli (Toner et al., 2009; Stark et al., 2010, 2013; Yassa et al., 2010a; Johnson et al., 2017; Yoder et al., 2017) and environments (Wilson et al., 2005; Robitsek et al., 2015). More recent ideas, however, contend that pattern separation and completion are not just relegated to the hippocampus, but also include cortical areas (Kent et al., 2016). Behavioral, imaging and lesion data strongly support this view (Bussey et al., 2006; Bartko et al., 2007a,b; Devlin and Price, 2007; McTighe et al., 2010; Graham et al., 2010; Behrmann et al., 2016). Therefore, in order to understand the complex mechanisms that underlie age-related cognitive decline, it is imperative to investigate the changes in cortical-hippocampal interactions in the context of behavior.

In young animals, CA3 neurons have unique activity patterns across different environments, such that the place fields in each environment are uncorrelated (Leutgeb et al., 2004; Vazdarjanova and Guzowski, 2004; Alme et al., 2014). This “remapping” of CA3 activity patterns does not occur to the same extent in aged rats (Wilson et al., 2005; Robitsek et al., 2015). While aberrant CA3 activity in old age has also been linked to a reduced ability to discriminate between similar objects (Toner et al., 2009; Stark et al., 2010, 2013; Yassa et al., 2010a; Johnson et al., 2017; Yoder et al., 2017), whether impaired sensory discrimination is associated with place field rigidity has not been determined. The current study behaviorally characterized young and aged rats on their abilities to learn to discriminate between two perceptually-similar objects (Johnson et al., 2017) over 10 days of behavioral testing. Rats were then subject to two distinct epochs of environmental exploration for 5 min. The pattern of neuronal activity was quantified across distal and proximal CA3 as well as the LEC by labeling *Arc* mRNA. *Arc* (activity-regulated cytoskeletal-associated gene; Lyford et al., 1995) in an immediate-early gene (IEG) with expression that does not require *de novo* protein synthesis (Lyford et al., 1995). Moreover, *Arc* is dynamically regulated by specific forms of patterned synaptic activity believed to underlie information storage (Cole et al., 1989). The transcription kinetics of *Arc* permit a cellular compartment analysis of temporal activity by fluorescence *in situ* hybridization (catFISH), allowing for the activity history of large samples of neurons across two episodes of behavior to be inferred from the subcellular location of *Arc* mRNA. Specifically, within 2 min of behavior-related activation of a neural ensemble, *Arc* RNA first appears in the nuclei of principal neurons at discrete sites of genomic transcription. After about 10 min, *Arc* RNA begins to leave the nucleus and accumulates in the cytoplasm, where it can be detected 15–30 min after activation (Guzowski et al., 2001). This approach was used to quantify the extent to which the active CA3 ensemble updated across different environments, and if this related to a rat's ability to discriminate between two similar objects. Furthermore, *Arc* catFISH was combined with retrograde tracing to identify those LEC neurons that project directly to CA3 (Conte et al., 2009a,b; Mesina et al., 2016). Importantly, analysis of the entorhinal cortex was restricted to the lateral area. We examined LEC, and not the medial entorhinal cortex (MEC), because available data indicate that LEC is more vulnerable in advanced age (Reagh et al., 2015; Reagh and Yassa, 2017) and the early stages of Alzheimer's disease (Khan et al., 2014), when compared to MEC. Moreover, the LEC, but not the MEC, is involved in discriminating between similar objects (Reagh and Yassa, 2014a), which was the task used in the current experiments to define behavioral groups.

Aged rats who performed poorly on a similar object LEGO object discrimination task (Johnson et al., 2017) showed a higher % of *Arc*-positive neurons in both CA3 and LEC projection neurons, but this did not relate to the extent that CA3 remapped. These data suggest that LEC contributes to the hyperactivity seen in CA3 of aged animals with cognitive deficits.

## Materials and methods

### Subjects

Twelve aged (24 months at time of arrival) and twelve young (4 months at time of arrival) male F344 × Brown Norway F1 rats from the NIA colony (Taconic) served as experimental subjects. Rats were housed individually in standard Plexiglas cages and maintained on a 12-h reversed light/dark cycle (lights off at 8:00 a.m.). All manipulations were performed during the dark phase of the cycle. Rats were given 1 week to acclimate to the facility and were handled for a minimum of 4 days before experimental procedures began. All rats initially participated in spontaneous (Ennaceur and Delacour, 1988) and cross-modal (Winters and Reid, 2010; Reid et al., 2012) object recognition tasks, followed by the water maze over 2 weeks. These data are part of another experiment and not shown here.

Following object recognition tasks, rats underwent stereotactic surgery for infusion of the retrograde tracer cholera toxin B-Alexa Fluor 594 (CTB) into the CA3 subregion of the hippocampus (see below). After surgery, rats were given 1 week to recover and then placed on a restricted feeding protocol to encourage appetitive behaviors. During food restriction, each rat was given 25 ± 5 g moist chow (Teklad LM-485, Harlan Labs) once daily and drinking water was provided *ad libitum*. Shaping began once rats reached ~85% of their baseline weights. Throughout the period of restricted feeding, rats were weighed daily and closely monitored to ensure they maintained an optimal body condition score of 2.5–3. If rats were not motived to perform for food rewards the baseline weight was readjusted by University of Florida veterinary staff. The body condition score was assigned based on the presence of palpable fat deposits over the lumbar vertebrae and pelvic bones (Ullman-Culleré and Foltz, 1999; Hickman and Swan, 2010). All procedures were in accordance with the NIH Guide for the Care and Use of Laboratory Animals and approved by the Institutional Animal Care and Use Committee at the University of Florida.

### Mircroinjection surgery

In order to identify lateral entorhinal cortical (LEC) cells with afferent projections to CA3, all rats received bilateral injections of the retrograde tracer cholera toxin subunit B (recombinant) Alexa Fluor 594 (CTB; ThermoFisher Scientific, cat # C22842) (Conte et al., 2009a,b) targeting the CA3 region (Paxinos and Watson, 2007). CTB (500 μg) was reconstituted and diluted to 1% in 50 μl of sterile 0.1 M PBS and stored at −20°C until surgery. Injections were administered with glass micro pipettes and a Nanoject II Auto-Nanoliter Injector (Drummond Scientific Company; Broomall, PA). Each hemisphere received three injections of 0.3 μl of CTB targeting CA3a (AP: −3.2 mm, ML: ±2.5 mm, and DV: −3.3 mm from dura), CA3b (AP: −3.2 mm, ML: ±2.0 mm, and DV: −3.3 mm from dura), and CA3c (AP: −3.2 mm, ML: ±1.5 mm, and DV: −3.3 mm from dura). Rats were given 1 week to recover from surgery before proceeding to behavioral testing on a LEGO® object discrimination task (Burke et al., 2011; Johnson et al., 2017).

### Object discrimination apparatus

**Figure 1:**
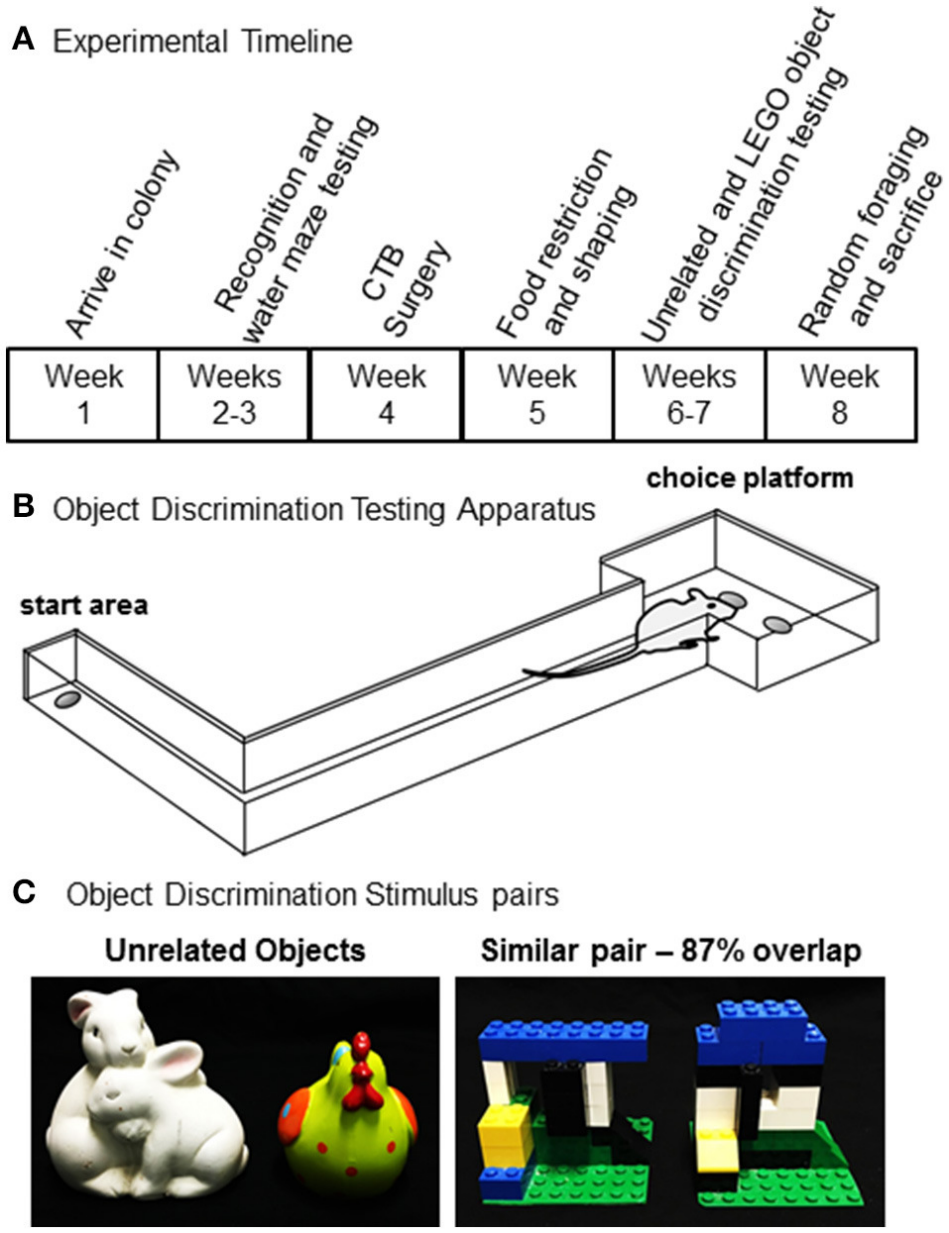
Experimental procedures. **(A)** Timeline for shaping, behavioral testing, and tissue collection for all rats used in the present experiment. **(B)** A schematic of the testing apparatus used for object discrimination test. **(C)** Unrelated (left) and similar (right) object pairs used for object discrimination testing.

Discrimination tasks were carried out in an L-shaped track bounded by a start area and choice platform, separated by an arm 84 × 10.2 cm with walls 6.4 cm in height (Figure 1B). The track was built out of plywood and sealed with waterproof black paint. In the start area, a single food well (2.5 cm diameter) was recessed 1 cm deep in the floor, 4 cm from the back wall. The choice platform measured 32 × 24 cm, also with walls 6.4 cm in height. Two food wells were centered relative to the platform entrance, 12 cm apart and positioned 7.6 cm from the back wall. The testing room was kept dimly lit with one white bulb angled to the wall near the start area and 2 red bulbs, one by the start area and one over the choice platform. This level of lighting was sufficient for the experimenters to comfortably see the objects. A white noise machine was kept on to reduce the impact of extraneous noise on performance.

### Unrelated and Lego® object discrimination testing

Discrimination sessions took place 7 days per week at approximately the same time each day. First, rats were habituated to the track for 3 days. Each rat was placed individually in the start area and allowed 10 min to retrieve scattered pieces of Froot Loop cereal (Kellogg's; Battle Creek, MI). Rats were then shaped to alternate between the start area and choice platform. For these sessions, one of the two food wells on the choice platform was baited with a piece of Froot Loop. Rats were required to leave the start area, traverse the length of the arm, retrieve the food reward, and return to the start area where a second food reward was provided. After shaping, rats were tested on an unrelated object discrimination task (Figure 1C, left panel) for 2 days to become familiar with the procedural aspects of discrimination testing. For this task, rats completed 32 trials in which one object was rewarded with a small piece of Froot Loop cereal in the food well below the object. The rat had to displace the object to retrieve the reward, and the side (left vs. right) of the rewarded object pseudorandomly varied from trial to trial. If the unrewarded object was moved the rat did not get to choose the alternate object and had to return to the start area to initiate the next trial. The rewarded object was counterbalanced across rats and age groups.

To characterize the extent that rats learned to discriminate between perceptually similar stimuli, a pair of objects were created from LEGO® blocks (Billund, Denmark; Figure 1C, right panel). Objects were matched for overall volume, shape, and texture, while systematically varying shared visible features. Both objects were built on a square piece of green plastic base sheet measuring 6.5 × 6.5 cm. Feature overlap between the two objects in each pair was estimated by percent of shared “pips” (that is, the unit in the LEGO matrix) in the 3-dimensional LEGO volume and was determined to be 87% volume and 63% visible features from the front face of the object (35 of 56 pips). As for the previous phase, rats completed 32 trials per day and the target object within each pair was counter-balanced across individual rats and age groups.

### Random foraging and sample collection

Three weeks post-CTB injection, animals underwent 5-min exploration epochs in two different 60 × 60 cm arenas, with a 20-min break in between. The foraging epochs were distinct in proximal visual cues within the arena and took place in two different testing rooms with unique distal visual cues, and rats were handled by different experimenters who conducted the random foraging procedures. To encourage foraging behavior, small pieces of Froot Loop cereal were randomly dropped around the arena and the rat would ambulate to retrieve the food. A webcam (Logitech; Newark, CA) recorded each exploration epoch using a custom software interface (Collector; Burke/Maurer Laboratories, Gainesville, FL). The data were later processed offline using custom written software in MATLAB (Mathworks, Natick, MA), to obtain the total distance moved and the proportion of the environment explored. Immediately following the second 5 min foraging epoch, rats were anesthetized in a glass bell jar with isoflurane vapors and decapitated with a rodent guillotine. Brains were rapidly extracted, hemisected, and flash frozen in chilled isopentane (~ −50°C). Brains were stored at −80°C until cryosectioning. In addition to the rats that randomly foraged, one young and one aged rat were sacrificed directly from the home cage as negative caged controls (Aged-CC and Young-CC). One hemisphere was processed for each rat and selection of the left or right hemisphere was counterbalanced across rats and age groups.

### Compartment analysis of temporal activity with fluorescence *in situ* hybridization (catFISH) and detection of *Arc*-positive neurons

Twenty-micron thick coronal sections of one hemisphere were cut on a cryostat (Microm HM550; Walldorf, Germany), and arranged on a slide so that tissue from both age groups and a caged control (sacrificed directly from the home cage) was represented on a single slide. During slicing all tissue was thaw-mounted on Superfrost Plus slides (Fisher Scientific; Hampton, NH) and stored at −80°C until FISH processing. Every 10th section was immediately DAPI stained to localize CTB injection sites and labeled projection neurons, and to ensure that the FISH procedures did not degrade the CTB signal. Twenty-four hours prior to FISH, tissue was moved to a −20°C freezer overnight to prepare for thawing. FISH was performed as previously described (Guzowski et al., 1999; Burke et al., 2005, 2012). Briefly, a commercial transcription kit and RNA labeling mix (Ambion REF #: 11277073910, Lot #: 10030660; Austin, TX) was used to generate a digoxigenin-labeled riboprobe with a plasmid template containing a 3.0 kb *Arc* cDNA (generously provided by Dr. A Vazdarjanova; Augusta University). Tissue was incubated with the probe overnight and *Arc* positive cells were detected with anti-digoxigenin-HRP conjugate (Roche Applied Science Ref #: 11207733910, Lot #: 14983100; Penzberg, Germany). Cyanine-3 (Cy3 Direct FISH; PerkinElmer Life Sciences, Waltham, MA) was used to visualize labeled cells and nuclei were counterstained with DAPI (ThermoFisher Scientific). Z-stack images with 1 μm optical sections were collected by fluorescence microscopy (Keyence; Osaka, Osaka Prefecture, Japan). For each rat, 6 images were taken from each region of interest (proximal CA3, distal CA3, and LEC) across 3–4 slides. All hippocampal CA3 images were collected between coordinates 3.0 and 4.0 mm posterior to bregma. All LEC images were collected between coordinates 5.6 and 6.5 mm posterior to bregma (Paxinos and Watson, 2007), which is where the majority of CTB+ LEC neurons were located. For all LEC images, the regions of interest were restricted to areas that had CTB labeling. Note, that one aged poor performing rat did not have any usable Arc data and was not included in any catFISH analyses.

Following image acquisition, the percent of *Arc* positive cells was determined by experimenters blind to animal age using ImageJ software. In order to exclude nuclei that were cut off by the edges of the tissue, only those cells that were visible within the median 20% of the optical planes in each z-stack were included for counting. All nuclei were first counted with the *Arc* channel off. When the total number of cells in the z-stack were identified, the *Arc* channel was turned on to classify cells as foci positive, cytoplasmic positive, both foci and cytoplasmic positive, or negative for *Arc*. A cell was counted as *Arc* foci positive if the fluorescent label could be detected within the nucleus at a transcription focus over 3 adjacent optical planes. *Arc* cytoplasm positive was assigned if the *Arc* signal surrounded at least half of the nuclear periphery over 3 adjacent planes. Cells that had both nuclear and cytoplasmic *Arc* where classified as both. Within the LEC, cells were classified as CTB-positive if they showed CTB channel signal surrounding at least half of the nucleus over 3 adjacent planes.

### Statistical analyses

For all dependent variables, normality of distributions was determined with a one-sample Kolmogorov–Smirnov test. In cases where the distribution was not normal, non-parametric tests were used to test for statistically significant differences.

When comparing the neural ensembles activated during two distinct epochs of behavior across different brain regions, it is helpful to parameterize the proportion of the four different subcellular distributions of *Arc* mRNA (negative, foci only, cytoplasm only, or both) with a value that can be used to compare activity patterns across different brain regions. The similarity score takes these four measured cell-labeling values and reduces them to a single value. Similarity scores were calculated for each rat that performed two epochs of behavior prior to sacrifice. A value of 0 indicates that two statistically independent cell populations were activated during the two epochs. A value of 1 indicates that an identical cell population was activated during the two epochs of behavior, while a value of -1 indicates that no cells were activated during both the first and second epochs of behavior. The similarity score was calculated as previously described (Vazdarjanova and Guzowski, 2004). Briefly, the similarity score is derived as follows: (1) epoch 1 (E1) active cells = proportion of cells with cytoplasmic only staining + proportion of cells with both cytoplasmic and foci staining. (2) Epoch 2 (E2) active cells = proportion of cells with foci only staining + proportion of cells with both cytoplasmic and foci staining. (3) p(E1E2) = proportion of cells active during E1 × proportion of cells active during E2. This is the probability of a neuron being active during both epochs, assuming that the two epochs of behavior activate statistically independent neural ensembles. (4) diff(E1E2) = proportion of cells with both foci and cytoplasmic staining − p(E1E2). This is the measure of deviation from the independence hypothesis. (5) Least epoch = the proportion of cells activated during the behavioral epoch with the smallest neural ensemble. (6) The similarity = diff(E1E2)/(least epoch − p(E1E2). Once similarity scores were calculated, the main effects of brain region and group were analyzed.

In addition to similarity score, the extent to which activated neural ensembles across regions deviated from a uniform random draw with replacement (URDWR) was quantified (Alme et al., 2010; Witharana et al., 2016). The URDWR model assumes that all cells have the same probability of activation in an environment of a given size, which can be represented by P1. If an animal is placed into a different environment each cell would again have an equal probability of being active (P2). Thus, the probability that a cell is active across both environments 1 and 2 is P1^*^P2. This can be used to calculate the total summed number of cells that one would expect to be active during exploration of both environments 1 and 2, or Px, based on the URDWR model with the following equation:

$$\begin{array}{l} \text{Px}=1-{\left(1-\text{P}1\right)}^{*}\left(1-\text{P}2\right) \end{array}$$

Px therefore represents the percent of cells that were active in two distinct environments, taking into consideration the cells that were active in both environments by chance. When the observed % of active neurons is less than Px, more cells were active in both environments than was expected by chance. When the observed % of active neurons is greater than Px, that means that if the cell was active in one environment it was less likely than expected by chance to be active in the second environment. In other words, activity in one environment biased the cell to be quiet in the next environment.

All statistics were run on the means for individual rats, such that sample size reflects the number of rats in each age group. When main effects were significant at the *p* < 0.05 level, additional comparisons between groups and analyses of the interaction effects were conducted with planned orthogonal comparisons or Tukey HSD *post-hoc* tests (SPSS software; SPSS Inc., Chicago, IL).

## Results

### Behavioral comparison in young and aged rats

**Figure 2:**
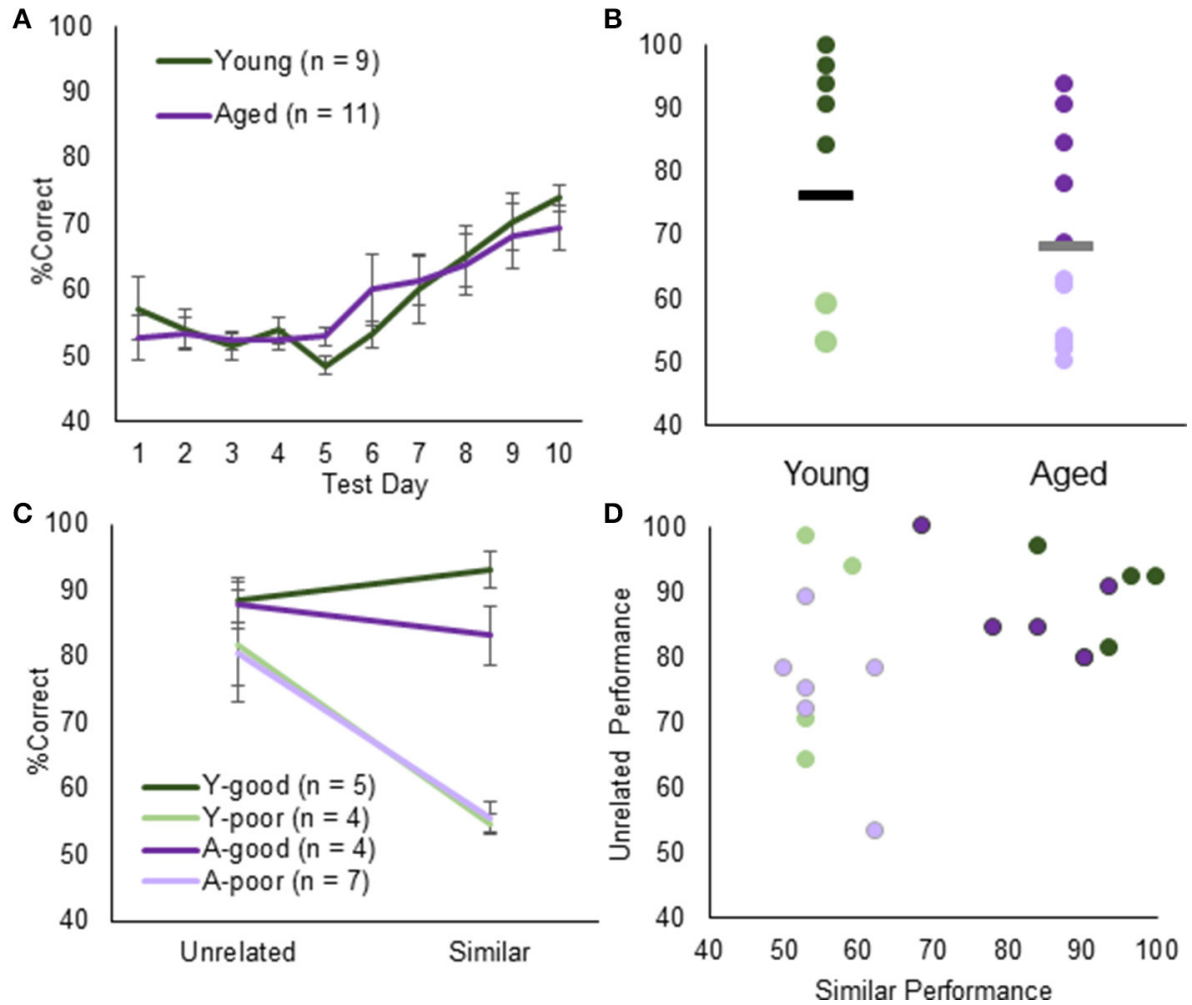
Object discrimination testing and performance. **(A)** Average % correct for young (green) and aged (purple) animals in discrimination testing. Overall, both groups acquired the task at the same rate. **(B)** When examining individual rat's performance on Day 10, however, the % correct follows a bimodal distribution across both groups. Using the K-means clustering algorithm, animals were segregated into young-good performers (green), young-poor performers (light green), aged-good performers (purple), and aged-poor performers (light purple). **(C)** This group division did not extend to either performance on the unrelated object discrimination. **(D)** Moreover, there was no systematic relationship between unrelated object discrimination and LEGO object discrimination. Therefore, impairments in the LEGO discrimination cannot be explained by procedural or sensorimotor deficits.

Once at an optimal body condition score was obtained (see above), rats were initially trained on an unrelated object discrimination problem in order to habituate them to the procedural aspects of object discrimination testing. All rats received 2 days of training on unrelated object discrimination (Figure 1C). Mean performance was ~84% correct responses (SEM = ±5.9) for the aged rats and 85% (SEM = ±4.2) for the young rats, which was not statistically different [*T*_(18)_ = 0.92, *p* = 0.37]. These data indicate that there was not an effect of age on the rats' abilities to learn to displace an object to retrieve a food reward, and to associate a particular object with a reward when the two objects to be discriminated were distinct (that is, did not share any features). Following discrimination testing with unrelated objects, rats completed 10 days of discrimination testing with LEGO® objects that shared 87% feature overlap (Burke et al., 2011; Johnson et al., 2017). The number of days of testing were limited to a total of 10 by the time frame in which the CTB signal in the brain begins to degrade (Wu et al., 2011). Figure 2A shows the percent correct responses as a function of test day for the young and aged rats. Previous research has shown that aged animals, relative to young, take significantly longer to learn to discriminate between similar objects when rats were tested until they reach a criterion of >81% correct responses for 2 consecutive days (Burke et al., 2011; Johnson et al., 2017). Although, the young rats made fewer errors than the aged rats on day 10 of testing, this was not statistically significant (*Z* = −0.53, *p* = 0.59; Mann–Whitney Test). While this may appear counter to prior research (Burke et al., 2011; Johnson et al., 2017), the effect is simply carried by testing duration as the current cohort of rats were not tested for a sufficient duration to detect reliable age differences in the number of trials required to learn which of the two LEGO® objects was rewarded. Overall, when comparing the % correct responses between unrelated object discrimination and LEGO® object discrimination, there was a significant effect of object type on performance (*Z* = −2.40, *p* < 0.02; Wilcoxon Signed-Rank Test), with all rats making fewer errors on the unrelated object discrimination compared to the LEGO® discrimination. The difference in % correct responses between unrelated and LEGO® object discriminations did not significantly vary with age (*Z* = −0.57, *p* = 0.57; Mann–Whitney Test).

Figure 2B shows the individual % correct responses on day 10 of testing for young and aged rats. Interestingly the distribution of performances in young and aged rats was not unimodal. The apparent bimodality across both groups was further supported by the fact that % correct responses on day 10 was not normally distributed (*p* < 0.05; one-sample Kolmogorov–Smirnov test). K-means clustering identified two clusters, one with a mean of 56.5 and another with a mean of 90.2. Seven aged rats were classified into the poor performer cluster and 4 aged rats in the good performer cluster. These animals were designated aged-poor performers (A-poor) and age-good performers (A-good), respectively. For the young rats, 4 were classified into the poor performer (Y-poor) cluster and 5 in the good performer (Y-good) cluster. This bimodal distribution of performances in both young and aged rats affords the ability to determine if the same neurobiological variables can explain behavioral differences across young and aged animals.

When performances across unrelated and similar overlap object discriminations were compared with A-good, A-poor and Y-poor and Y-good performers (Figure 2C) there was a significant effect of group on the performance difference between discrimination conditions [${\text{Chi}}_{\left(3\right)}^{2}$ = 8.48, *p* < 0.05; Kruskal–Wallis Test]. This was due to all groups performing similarly on the unrelated discrimination problem [${\text{Chi}}_{\left(3\right)}^{2}$ = 5.75, *p* = 0.13; Kruskal–Wallis Test], while the Y-poor and the A-poor groups had significantly fewer correct trials on the similar object discrimination compared to the other two groups (*p* < 0.001 for all comparisons). Importantly, there was not a significant correlation between individual rats' performance on the unrelated and similar object discrimination tasks [*r*_(19)_ = 0.35, *p* = 0.13; Figure 2D], suggesting that a reduced ability to learn the procedural aspects of object discrimination and/or sensorimotor impairments did not influence performance on the LEGO discrimination task.

**Figure 3:**
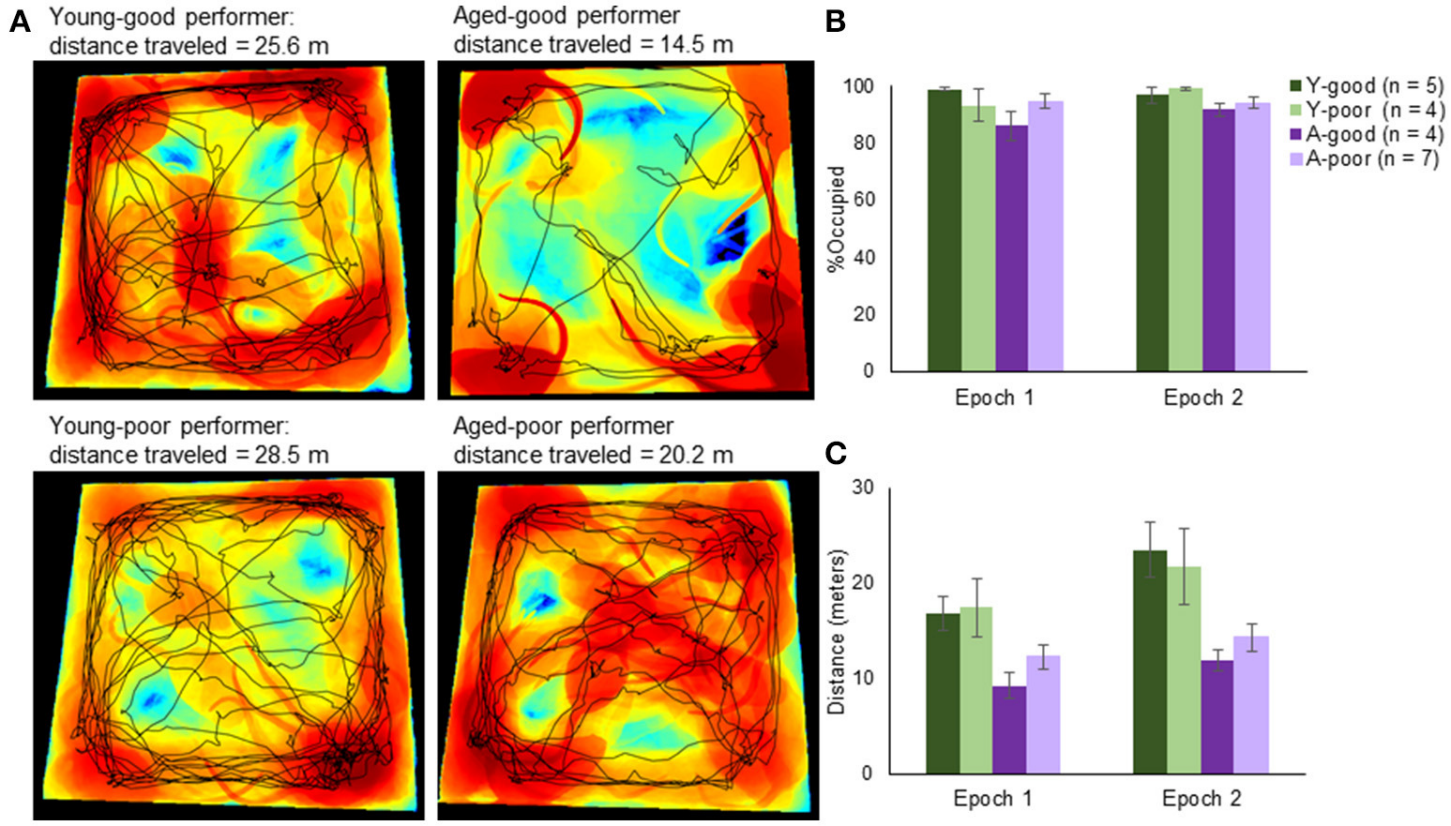
Arena exploration performance. **(A)** Representative occupancy heat maps and travel paths across age and behavioral group. Occupancy maps were created using custom computer vision software written in MATLAB. Briefly, background subtraction allowed a segmented representation of the total area that the rat occupied within a single frame (from nose to tail and everything in between). Black areas represent areas unexplored by the animal, blue is low occupancy and red is high occupancy. Superimposed on top of the occupancy heat map is the exploration path (black line). **(B)** Quantifying the total area across rats, there was no significant difference in novel area coverage across groups. **(C)** There was, however, a significant difference in the total distance traveled with rats traveling farther in the second epoch relative to the first. Moreover, consistent with slower ambulation velocities with age, the aged animals had lower cumulative path-lengths.

At the conclusion of discrimination testing, rats randomly foraged for food rewards in two different novel arenas placed in distinct environments during two 5-min epochs. During each 5-min epoch, rats voluntarily ambulated for small pieces of Froot Loop cereal. In order to provide orthogonal conditions, the rooms, foraging arenas and experimental handlers differed between epochs 1 and 2. A 20-min delay was imposed between each epoch in which the rat remained in their home cage. Figure 3A shows representative paths and occupancy heat maps obtained from the exploration epochs for young (left) and aged rats (right). For all rats, the percent of the environment occupied and the distance traveled was quantified and compared between the four groups and epochs 1 and 2 (Figure 3B). There was not a significant difference in percent of the arena occupied between epoch 1 (arena A) and epoch 2 (arena B) (*Z* = -0.64, *p* = 0.53; Wilcoxon Signed-Rank Test). Additionally, there was not a significant effect of group on arena coverage [${\text{Chi}}_{\left(3\right)}^{2}$ = 6.16, *p* = 0.10; Kruskal–Wallis Test]. Therefore, the total amount of novel area explored was similar across all groups.

The mean distance traveled (Figure 3C) in epoch 1 was 13.7 and 17.5 m in epoch 2, and these were normally distributed for both young and aged rats (*p* > 0.1; One-sample Kolmogorov–Smirnov Test). When the distance traveled within each epoch was compared across arenas, there was a significant effect of epoch [*F*_(1, 16)_ = 14.54, *p* < 0.01]. This was due to the rats walking a greater distance in the second epoch, likely because they became accustomed to the random foraging procedures. There was also a significant effect of group on distance traveled [*F*_(1, 16)_ = 6.37, *p* < 0.01]. *Post-hoc* analyses indicated that the good and poor performing young rats traversed a similar distance (*p* = 0.99; Tukey), as did the A-good and A-poor rats (*p* = 0.71). In contrast, the A-good rats traversed significantly fewer meters compared to both groups of young rats (*p* < 0.05 for both comparisons). There was not a significant interaction effect of group and distance traversed [*F*_(1, 16)_ = 1.09, *p* = 0.38], indicating that the aged rats moved less in both epochs compared to young. This was likely due to the slower ambulation velocities typically observed with age. This factor was considered with respect to the levels of *Arc* expression (see below). Notably, while the aged rats moved slower, they did explore similar portions of the arenas compared to the young rats.

### *Arc* expression patterns in CA3 neural ensembles

**Figure 4:**
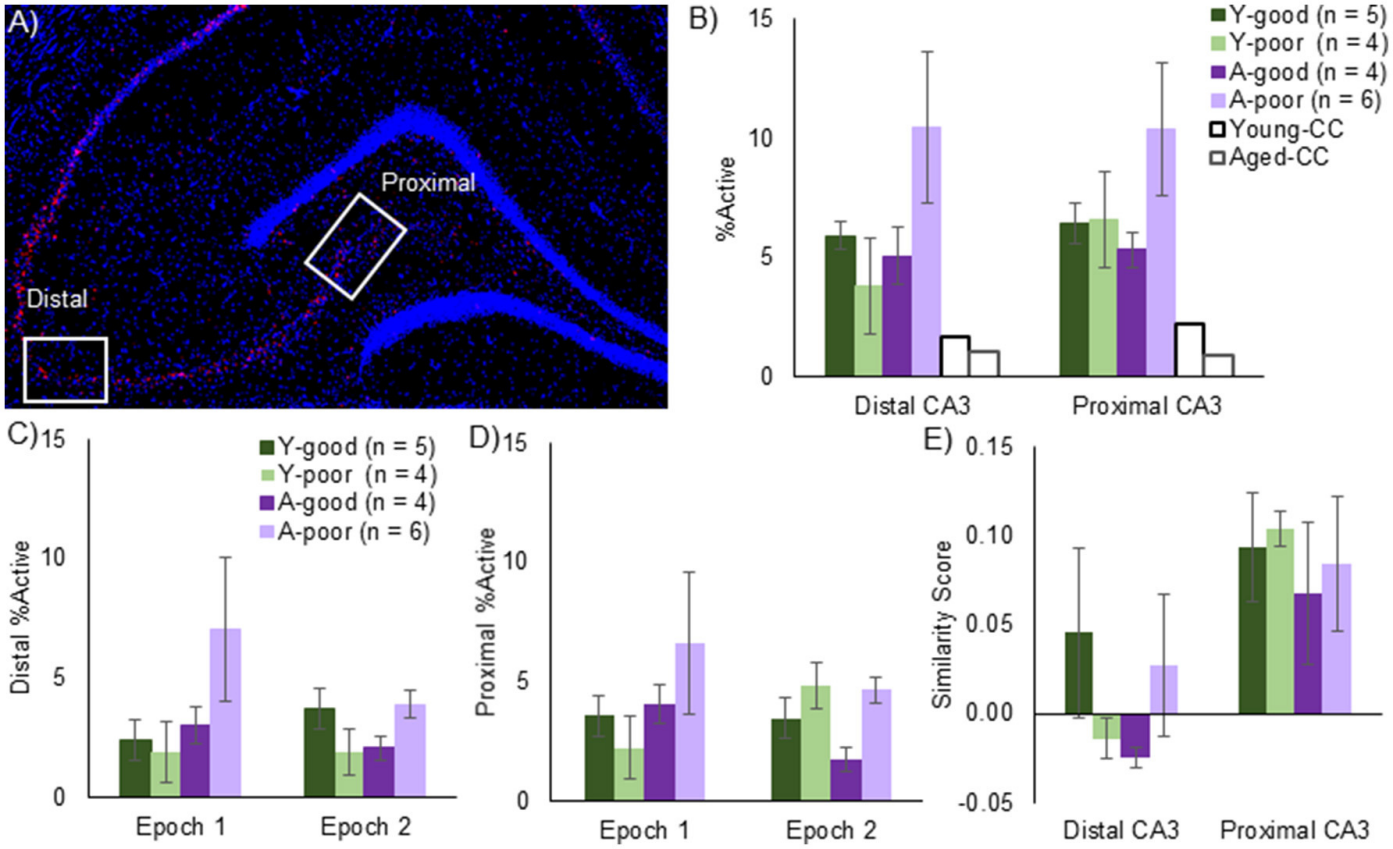
CA3 Arc expression. **(A)** Mosaic image of *Arc* expression across the hippocampus. Z-stack images were taken at 40x magnification from the regions in the white rectangles, denoted as either distal or proximal CA3. **(B)** Total % of *Arc* positive neurons across both epochs for all behavioral groups and a young and aged caged control (CC). Note that, while there was no effect of CA3 subregion on the proportion of active neurons, aged-poor performing animals had significantly higher overall *Arc* positive cells supporting the hypothesis that CA3 hyperexcitability underlies cognitive deficits. Effect of epoch on distal CA3 **(C)** and proximal CA3 **(D)** *Arc* expression. Epoch did not significantly affect *Arc* expression in either region, although the aged-poor performing rats again demonstrated significantly higher proportion of active neurons. **(E)** Traditional Similarity score across CA3 subregions. In order to examine the conventional idea of remapping, in which neurons are randomly assigned to an environment, similarity scores were calculated. The similarity, or more specifically, the lack thereof was consistent across age-groups. The distal CA3 region, however, exhibited less overlap relative to the proximal region. Note, that one aged poor performing rat did not have any usable *Arc* data and was not included in any catFISH analyses.

The average number of cells captured per image within CA3 was 51.15 for the young rats and 48.21 for the aged rats, which was not statistically different [*T*_(18)_ = 0.55, *p* = 0.52]. Cell number also did not vary significantly between any of the behavioral groups [Y-good, Y-poor, A-good, A-poor; *F*_(3, 16)_ = 0.97, *p* = 0.43], which is consistent with stereological counts showing no loss of CA3 neurons with age (Rapp and Gallagher, 1996). Figure 4A shows a stitched image of the hippocampus from an aged rat (taken at 10X magnification) labeled for *Arc* mRNA (red) and counterstained with DAPI (blue). The white rectangles indicate the distal and proximal regions of CA3 from which z-stack images were acquired at 40X magnification. When the total % of CA3 cells that expressed *Arc* (Figure 4B) were compared across distal vs. proximal CA3 for the different groups, there was not an effect of region [*F*_(1, 32)_ = 0.34, *p* = 0.56], which is consistent with a previous report (Marrone et al., 2014). Behavioral group, however, did have a significant effect on the percent of cells that were positive for *Arc* mRNA [*F*_(3, 32)_ = 3.16, *p* < 0.05]. Two planned orthogonal contrasts were run in order to test for specific differences between groups. A simple contrast in which the A-poor rats were compared to every other group indicated that these animals had significantly more CA3 neurons that expressed *Arc* relative to the A-good and Y-good performing animals (*p* < 0.02 for both comparisons; corrected α = 0.05/2). There was also a non-significant trend for the A-poor group to have more *Arc*-positive cells than the Y-poor performers (*p* = 0.04; corrected α = 0.05/2). In contrast to the A-poor group, the Y-poor performers did not have significantly different percentages of *Arc* positive cells compared to either the Y-good performers or A-good group (*p* > 0.64 for both comparisons). These data suggest that enhanced activity in CA3 can explain behavioral variability on a discrimination task in aged animals, but not in young animals.

When the percent of neurons expressing *Arc* was compared across epochs for distal (Figure 4C) and proximal (Figure 4D) CA3 with a repeated-measures ANOVA with the within subject factor of epoch and the between subjects factors of region (distal vs. proximal) and behavioral group (Y-good, Y-poor, A-good, and A-poor), there was not a significant effect of epoch [*F*_(1, 32)_ = 0.75, *p* = 0.39] or region [*F*_(1, 32)_ = 0.66, *p* = 0.42]. There was, however, a significant effect of group [*F*_(3, 32)_ = 3.19, *p* < 0.05] with the A-poor rats having significantly more cells expressing *Arc* compared to the other groups (*p* < 0.05 for all comparisons). None of the interactions between factors reached statistical significance (*p* > 0.2 for all comparisons). These data provide further support that the A-poor rats had more cells in CA3 that expressed *Arc* during behavior. Importantly, there was not a significant correlation between distance traversed in epoch 1 and epoch 2 and the percent of cells that were Arc positive in either distal [*r*_(19)_ = −0.20, *p* = 0.40; *r*_(19)_ = 0.10, *p* = 0.69, respectively], or proximal CA3 [*r*_(19)_ = −0.13, *p* = 0.60; *r*_(19)_ = 0.11, *p* = 0.67, respectively]. These data suggest that group differences in total distance traveled could not account for the increased proportion of CA3 cells expressing *Arc* in the A-poor performing rats.

The degree to which neuronal ensembles activated during two distinct epochs of behavior are uncorrelated can be quantified by calculating a similarity score (Vazdarjanova and Guzowski, 2004), which converts the subcellular distribution of Arc (foci only, cytoplasm only, or both) to a single value that provides a quantitative measure of the population overlap. A low similarity score is consistent with uncorrelated neural ensembles such that the overlapping activity (cells with both foci and cytoplasmic labeling) does not exceed chance levels. Conversely, a high score suggests that the same population of cells was active during both epochs of behavior. Figure 4E shows that mean similarity scores for proximal and distal CA3 for the different behavioral groups. Overall, a repeated-measures ANOVA with the within subjects factor of position along the transverse axis of CA3 (distal vs. proximal) and the between subjects factor of behavioral group indicated that the similarity score was significantly lower for distal CA3 compared to proximal CA3 [*F*_(1, 16)_ = 11.15, *p* < 0.01]. Behavioral group did not have a significant effect on similarity score [*F*_(3, 16)_ = 0.64, *p* = 0.60], and did not significantly interact with position on the transverse axis [*F*_(1, 16)_ = 0.44, *p* = 0.73]. These data indicate that when rats explored two distinct environments that were both novel, all groups showed recruitment of distinct neural populations in CA3.

Recent data have shown that a subset of cells in a hippocampal neural ensemble activated during an episode of exploration may be used redundantly when the environment changes, such that a greater number of cells have place fields in both environments than would be expected by chance (Alme et al., 2010; Mizuseki and Buzsáki, 2013; Buzsáki and Mizuseki, 2014; Witharana et al., 2016). This is believed to reflect skewed, high firing rate cells that are more likely to remain active across multiple environments (Mizuseki and Buzsáki, 2013; Buzsáki and Mizuseki, 2014; Witharana et al., 2016). One way to examine the extent that neural ensembles follow a skewed excitability distribution rather than adhere to a uniform random draw with replacement (URDWR) model is to compare the expected percent of active cells across multiple epochs of exploration (Px) relative to the observed percent of cells. In this case, the observed percent of activated cells in the two environments would be the sum of %neurons with Arc in the nucleus + %neurons with Arc in the cytoplasm + %neurons with Arc in both the nucleus and the cytoplasm. When the observed % of active neurons is less than Px, more cells were active in both environments than was expected by chance. When the observed % of active neurons is greater than Px, that means that if the cell was active in one environment it was less likely than expected by chance to be active in the second environment. In other words, activity in one environment biased the cell to be quiet in the next environment.

**Figure 5:**
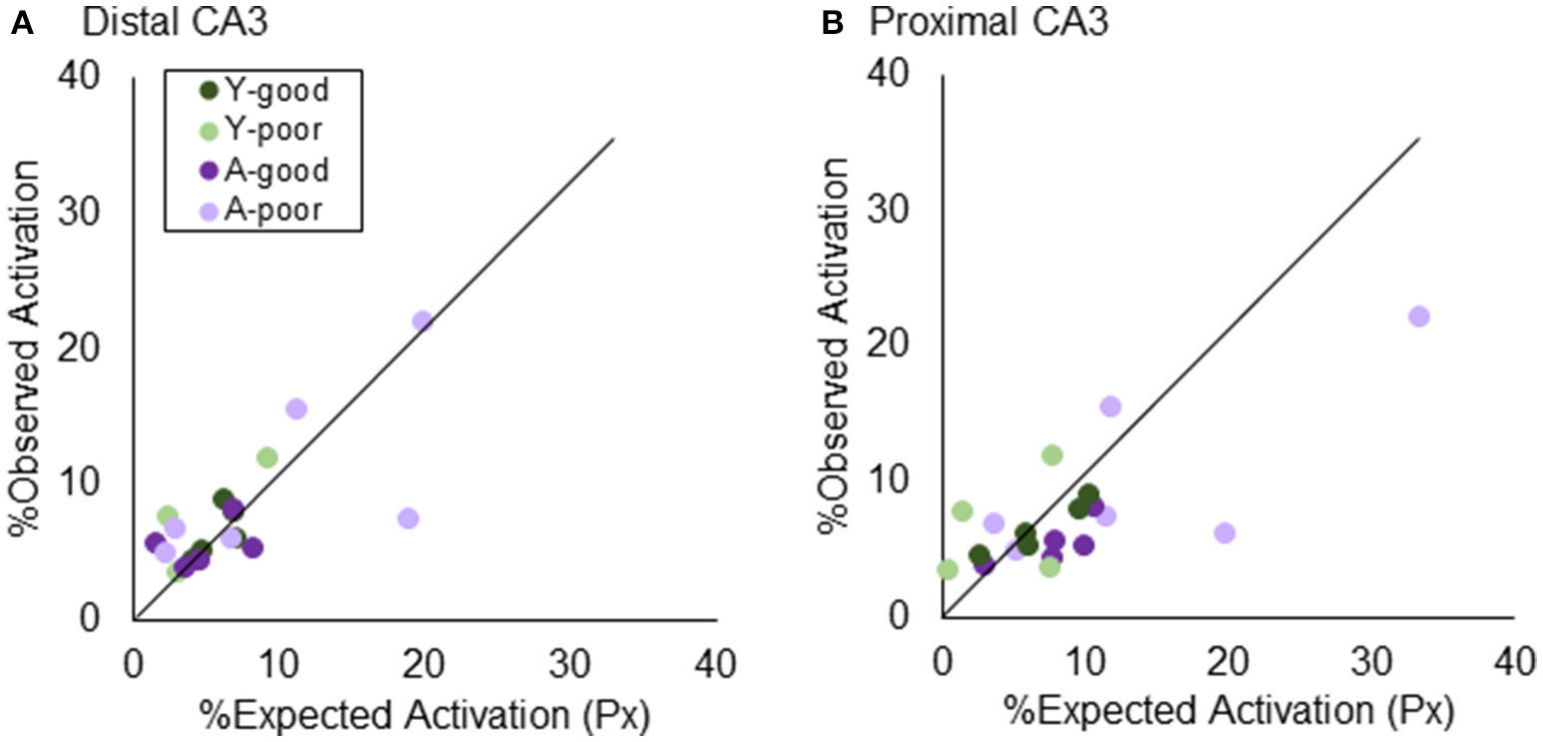
Testing “Uniform Random Draw with Replacement” in CA3. The similarity score assumes that neuronal activity patterns in an environment are randomly selected. Prior results using a thresholding algorithm found that CA3 “under-recruited” neurons. Using stereologically-based quantification, both **(A)** distal and **(B)** proximal CA3 did not significantly deviated from the URDWR model.

Figure 5 shows the observed percent of cells that were positive for *Arc* after two epochs of exploration, plotted against the expected activation based on activity in epoch 1 and epoch 2 under the uniform random draw with replacement model for distal (Figure 5A) and proximal CA3 (Figure 5B). Observed activation did not significantly differ from expected activation for either distal (*Z* = −0.63, *p* = 0.52; Wilcoxon Signed-Rank Test) or proximal CA3 (*Z* = −0.93, *p* = 0.35; Wilcoxon Signed-Rank Test). Furthermore, the deviation from expected activation was not significantly different between the four behavioral groups in either distal [${\text{Chi}}_{\left(3\right)}^{2}$ = 0.81, *p* = 0.85; Kruskal–Wallis Test] or proximal CA3 [${\text{Chi}}_{\left(3\right)}^{2}$ = 5.62, *p* = 0.13; Kruskal–Wallis Test]. These data indicate that under the behavioral parameters of the current experiment there was not a detectable difference in the probability that a CA3 neuron would be active across multiple environments between young and aged rats.

### *Arc* expression patterns in lateral entorhinal cortical projection neurons to CA3

**Figure 6:**
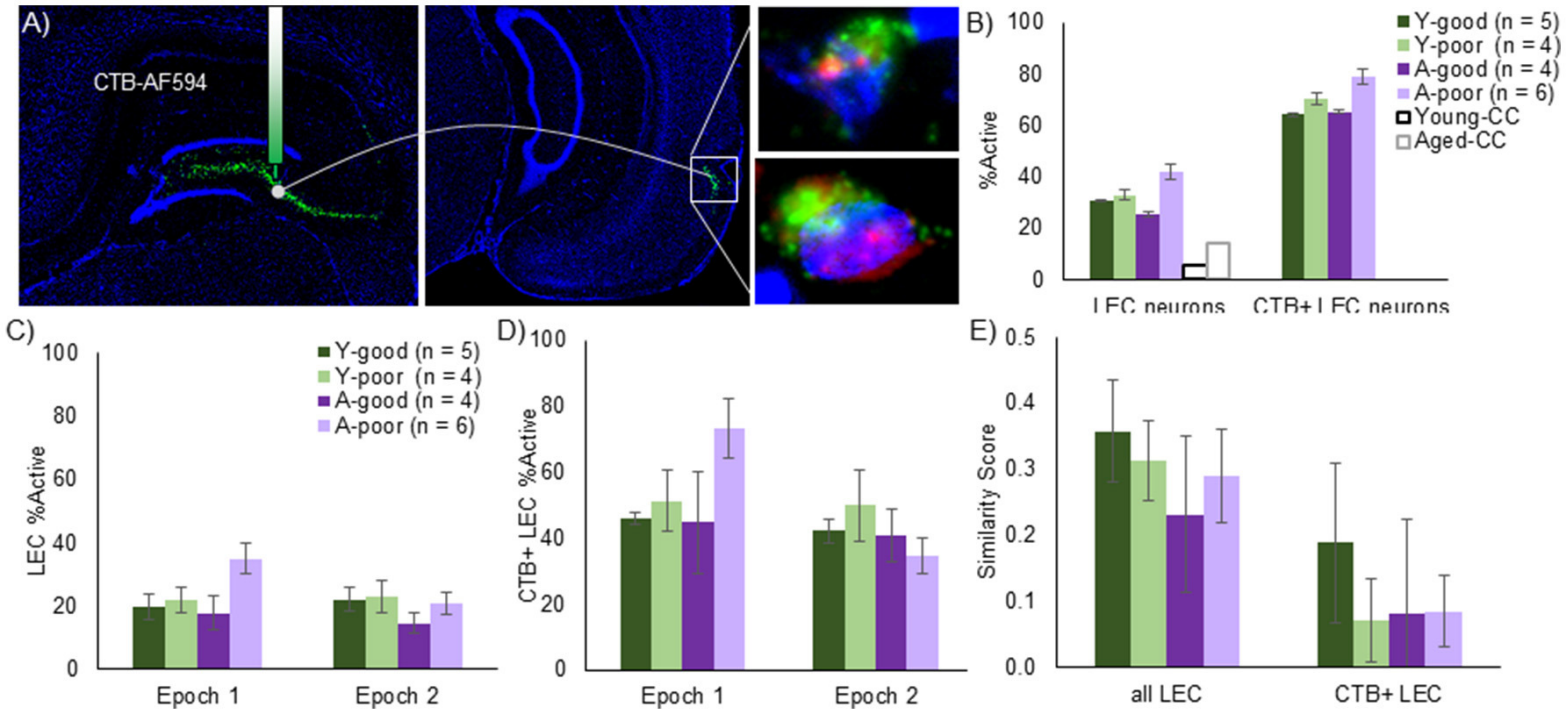
LEC Arc expression in the context of anatomical projections. **(A)** Mosaic images and schematic of CTB/*Arc* procedure. The retrograde tracer, CTB, was infused into the CA3 regions and retrogradely transported to cell bodies of LEC neurons that project to this region. Following *in situ* hybridization, Layer II neurons in the LEC could be identified that are co-labeled with tracer as well as positive for *Arc* expression. The right panels show representative CTB+ cells with *Arc* in the nucleus (top) and *Arc* in both the nucleus and the cytoplasm (bottom). **(B)** Using this approach, LEC neurons with projections into the CA3 region (CTB+ LEC neurons) can be analyzed relative to the entire LEC population. The % of cells that expressed *Arc* expression was significantly higher number for the LEC→CA3 projection neurons compared to all layer II LEC neurons and this did not significantly interact with age. When taking each epoch into consideration, however, the proportion of *Arc* positive neurons were higher in the first epoch for the entire population **(C)** as well as the LEC→CA3 projection neurons **(D)**. Moreover, the age-poor performing group exhibited a significantly higher % of *Arc* positive cells in epoch 1 relative to the other groups. **(E)** Traditional Similarity score for all LEC neurons and LEC→CA3 projection neurons. The LEC→CA3 projection neurons had a significantly lower similarity score across epochs relative to all layer II LEC neurons.

The observation that A-poor, but not A-good, rats have more active CA3 ensembles raises the question of whether the afferent input to CA3 contributes to this hyperactivity. To examine the possibility that entorhinal input to the CA3 contributes to the larger proportion of CA3 cells activated during behavior, the retrograde tracer cholera toxin B conjugated with Alexa Fluor 594 (CTB) was injected into CA3 of all rats 21 days before sacrifice. Layer II LEC neurons that were positively labeled for CTB were identified as projecting to CA3 (Figure 6A). The average number of LEC cells counted per image was 40.94 for the young rats and 37.79 for the aged rats, which was not statistically different [*T*_(16)_ = 1.0, *p* = 0.33]. Of these cells, the average number of CTB positive neurons was 8.26 for the young rats and 8.20 for the aged rats, which was also not significantly different [*T*_(16)_ = 0.04, *p* = 0.97]. Total LEC cell number [*F*_(3, 16)_ = 0.45, *p* = 0.72] and CTB positive cell number [*F*_(3, 16)_ = 1.41, *p* = 0.28] also did not vary significantly between any of the behavioral groups (Y-good, Y-poor, A-good, A-poor). These data are consistent with stereological counts showing no loss of LEC neurons with age (Rapp et al., 2002). Critically, these data also indicate that the loss of perforant path fibers that is well documented with age (Barnes and McNaughton, 1980; Foster et al., 1991; Yassa et al., 2010b) does not impact the probability of the tracer being transported back to the cell body. Thus, LEC neurons continue to send fibers to the hippocampus even though individual cells may send fewer fibers in old animals compared to young. Alternatively, it is conceivable that the fiber loss in old age is predominately from cells in the MEC.

The percent of cells that expressed *Arc* was significantly higher when the LEC neuron population was restricted to those neurons that project to CA3 compared to all layer II LEC cells [Figure 6B; *F*_(1, 15)_ = 261.90, *p* < 0.001]. The increased likelihood of a cell being active in projection neurons did not significantly interact with behavioral group [*F*_(1, 15)_ = 0.33, *p* = 0.80]. Moreover, when the total percent of all LEC cells that expressed *Arc* was compared across the different behavioral groups, there were no significant differences across groups [Figure 6B; *F*_(3, 15)_ = 1.83, *p* = 0.19]. However, when the percent of neurons expressing *Arc* was compared across epochs for all layer II LEC neurons (Figure 6C) and for LEC to CA3 projection neurons (Figure 6D) with repeated-measures ANOVA with the within subject factor of epoch and the between subjects factor of behavioral group (Y-good, Y-poor, A-good, and A-poor), there was a significant effect of epoch for all LEC neurons [*F*_(1, 15)_ = 4.53, *p* < 0.05] and for the LEC to CA3 projection neurons [*F*_(1, 15)_ = 5.58, *p* < 0.05] such that the proportion of *Arc* positive LEC neurons was higher in the first epoch relative to the second. There was also a significant interaction of behavioral group and epoch for all LEC neurons [*F*_(3, 15)_ = 5.85, *p* < 0.01], and for CA3 projecting LEC neurons [*F*_(3, 15)_ = 4.01, *p* < 0.05]. *Post-hoc* analysis indicated that this was due to the A-poor group having a significantly higher percent of LEC cells expressing *Arc* compared to the other groups during epoch 1 (*p* < 0.05 for all comparisons), but not during epoch 2 (*p* > 0.28 for all comparisons). The same pattern in the data were observed for all layer II LEC cells when the analysis was restricted to CA3 projecting neurons.

To examine the extent that LEC neurons update their activity patterns when the environment changes, a similarity score was calculated from the distribution of *Arc* in all layer II LEC neurons and in the CA3 projecting neurons (Figure 6E). A repeated-measures ANOVA with the within subject factor of LEC neuron population (all vs. CA3 projecting) indicated a significant effect of LEC neuron population on the similarity score [*F*_(1, 15)_ = 28.93, *p* < 0.001]. Specifically, LEC neurons that project to CA3 are significantly more likely to have non-overlapping populations of neural activity between two epochs of exploring different novel environments than the general population of layer II LEC neurons. This did not significantly interact with behavioral group [*F*_(3, 15)_ = 0.06, *p* = 0.81] indicating that, similar to the A-good and young animals, A-poor rats had a new population of cells active when the environment changed.

**Figure 7:**
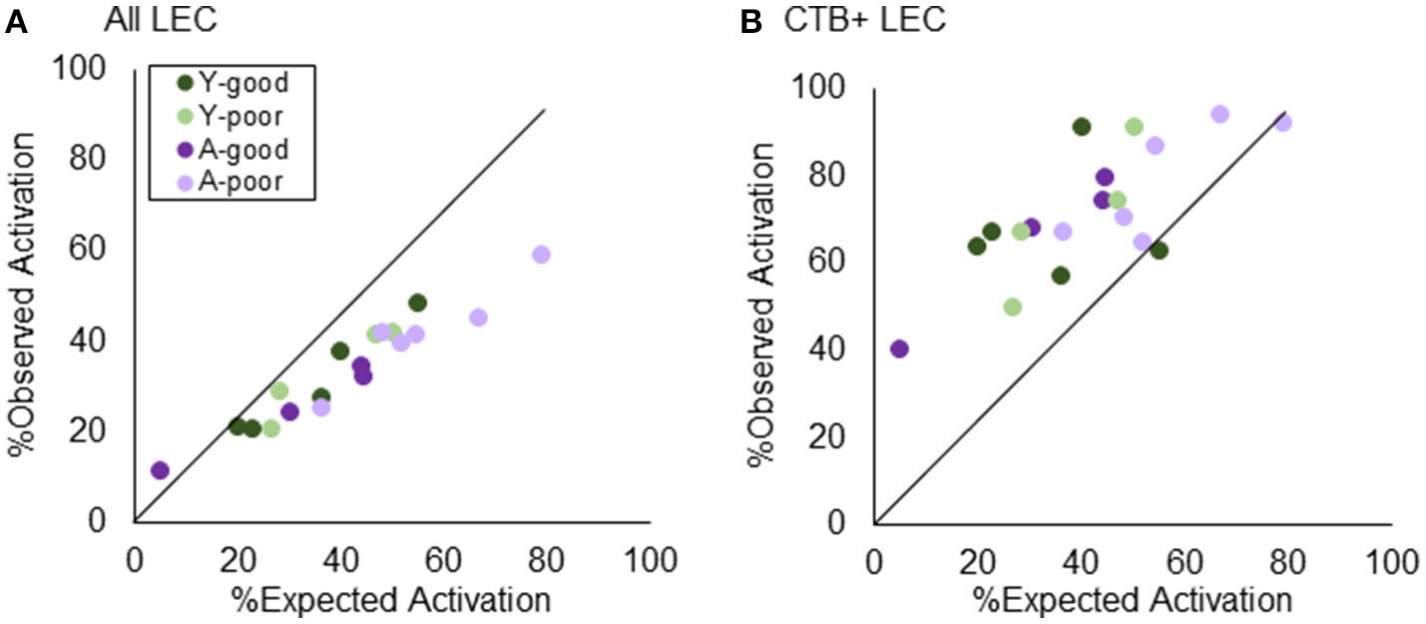
Testing “Uniform Random Draw with Replacement” in LEC. Revisiting the nature of neurons being utilized redundantly across environments, layer II LEC neurons were quantified in terms of their observed vs. expected distribution of active cells. **(A)** When the analysis encompassed all layer II LEC neurons, the expected activation outweighed the observed, suggesting that more LEC neurons are activated during both epochs of exploration than what is expected by chance. **(B)** Restricting the analysis to the LEC→CA3 projection neurons, however, demonstrates the opposite effect. The observed % of active neurons outweighs the expected, suggesting a dissimilar pattern across environments beyond what has normally been explained as “chance.”

Figure 7 shows the percent of cells in LEC activated during two epochs of exploration, plotted against the predicted levels of *Arc* positive cells for all LEC cells (Figure 7A) and for CTB+ LEC cells (Figure 7B). When all LEC cells were examined, the observed percent of activated cells was significantly less than what was expected under the uniform random draw with replacement model (*Z* = −3.54, *p* < 0.001; Wilcoxon Signed-Rank Test). This suggests that more LEC neurons are likely to be activated during both epochs of exploration than what is expected by chance, which is consistent with a previous report (Mizuseki and Buzsáki, 2013). The skewed activity distribution in LEC also significantly differed by behavioral group [${\text{Chi}}_{\left(3\right)}^{2}$ = 8.30, *p* < 0.05; Kruskal–Wallis Test] such that the A-poor rats showed a significantly larger deviation from predicted values relative to the other groups. This is likely due to the tendency for a greater proportion of A-poor LEC neurons to be active in epoch 1 than epoch 2. Interestingly, a different pattern in the data emerged when the analysis was restricted to the CTB+ LEC neurons. For these cells, the observed percent of activated cells was significantly greater than what was predicted under the uniform random draw with replacement model (*Z* = 2.36, *p* < 0.05; Wilcoxon Signed-Rank Test). This deviation from expectation did not significantly differ between the four behavioral groups [${\text{Chi}}_{\left(3\right)}^{2}$ = 1.95, *p* = 0.58; Kruskal–Wallis Test]. Together these data indicate that CA3-projecting LEC neurons have different activity dynamics than the larger population of LEC neurons, which favor the recruitment of orthogonal neural ensembles across different behavioral episodes.

### Comparison of CA3 and LEC activity patterns

The total percent of cells positive for *Arc* in CA3 was significantly more sparse compared to LEC neurons that project to CA3 [*F*_(1, 15)_ = 329.05, *p* < 0.001], which did not interact significantly with behavioral group [*F*_(3, 15)_ = 1.21, *p* = 0.34]. When the similarity score was used to compare the extent that CA3 cells and CA3-projecting LEC cells have independent populations of active neurons during the two different epochs of exploration, there was not a significant effect of region on similarity score [*F*_(3, 15)_ = 2.32, *p* = 0.12]. These data suggest that the probability of cells being active in both environments did not differ between LEC projection neurons and CA3. Moreover, for the young rats, the similarity score for LEC projection neurons was negatively correlated with the similarity score for proximal CA3 neurons [*r*_(8)_ = −0.83, *p* < 0.01], and positively correlated with the similarity score for distal CA3 neurons [*r*_(8)_ = 0.79, *p* < 0.05]. In both cases, however, this effect appeared to be carried by one outlier and when that data point was removed, these relationships were no longer significant (*p* > 0.3 in both cases). One difficulty with examining correlations between CA3 and LEC similarity scores is that they cluster between 0 and 0.1, which does not offer parametric space for detecting significant relationships. Future experiments will need to examine the possibility of this relationship using behavior procedures that lead to more variable amounts of overlap between active neuronal ensembles. In the aged rats, there was not a significant relationship between similarity scores calculated from projection neurons in LEC and proximal [*r*_(9)_ = −0.53, *p* = 0.11] or distal CA3 [*r*_(9)_ = −0.09, *p* = 0.81].

## Discussion

The current experiments were initially designed to measure the extent to which the inability of aged CA3 place fields to update in a new environment could be accounted for by sensory discrimination deficits. Aged and young rats were pre-characterized on a LEGO® object discrimination task over 10 days of testing to quantify sensory discrimination abilities (Figures 1, 2). After discrimination testing, rats randomly foraged in two different novel environments for 5 min each, separated by 20 min. This enabled the neurons activated in each environment to be determined by identifying the subcellular location of *Arc* mRNA. The original hypothesis was that those animals with the greatest discrimination impairments would be the same animals in which CA3 ensembles would not remap across environments. We also predicted that this would be related to rigid activity patterns in the LEC neurons that project to CA3. Surprisingly, all rats showed orthogonal ensembles of active cells in CA3 and LEC across the two novel environments.

Importantly, in both age groups, there was a subset of rats that did not learn to discriminate between two perceptually similar objects by day 10. A novel finding from this work is that only the aged-impaired rats had a larger percent of principal cells in both CA3 and LEC that were positive for the immediate-early gene *Arc* following two epochs of random foraging in different novel environments. In contrast, the young rats that performed poorly on the LEGO object discrimination task had comparable percentages of cells that were positive for *Arc* to the young rats that were good performers on the task (Figure 4). Thus, distinct neurobiological mechanisms account for behavioral variability in young vs. old age. The fact that the poor performing aged rats on the object discrimination task where the same animals that had an over active CA3 during exploration of a novel environment suggests that these animals would also have CA3 hyperactivity during discrimination behavior. This prediction is consistent with human fMRI data showing an increased BOLD signal in CA3/dentate gyrus during the mnemonic similarity task in aged-impaired individuals (Yassa et al., 2010a, 2011). Future research will need to measure Arc expression in CA3 during the LEGO object discrimination task to test this hypothesis, however.

The observation that aged, but not young, poor performing rats have a higher proportion of active CA3 cells during behavior leaves the question as to whether the poor-performing young rats are expressing a behavioral phenotype that predicts age-related impairments. Longitudinal studies have shown that the young animals with more impulsive traits are more likely to develop cognitive deficits in advanced age (Dellu-Hagedorn et al., 2004). During acquisition of the LEGO object discrimination task rats begin with a bias in which they chose an object on a particular side, regardless of the object identity (Johnson et al., 2017). Rats need to overcome the impulse to choose a side in order to learn to select the target (Lee and Byeon, 2014; Hernandez et al., 2015; Johnson et al., 2017). It is conceivable that young rats with more impulsive behavior are predisposed to develop neurobiological dysfunction that contribute to behavioral decline.

Wilson et al. (2005) were the first to report higher CA3 firing rates in aged rats with impairments on the water maze. It is difficult to infer the proportion of overall population activity in this study, however, as only 1–10 cells were recorded simultaneously within a behavioral epoch. More recent data have also reported higher CA3 firing rates in aged rats during exploration of a novel environment (Robitsek et al., 2015), but did not specifically analyze for the proportion of cells that were active. Therefore, the data presented here provide a novel extension from the previous literature in which the higher rates in aged CA3 neurons are accompanied by a larger proportion of cells that are active during behavior. It is conceivable that with elevated firing rates, CA3 principal recruit additional neurons into the active ensemble through autoassociative projections. Interestingly, higher percentages of aged, compared to young, CA3 neurons that were active during random foraging were more evident during the first epoch. This may be analogous to the data reported in Robitsek et al. (2015) in which firing rates of old CA3 cells were only elevated relative to young when the environment was novel, but not familiar. From this perspective, the present data suggest that the aged impaired rats did not recognize that second environment was novel despite being conducted in different rooms with different experimenters.

Consistent with the electrophysiological data from animal models, high-resolution human fMRI data have reported a higher BOLD signal in the dentate gyrus/CA3 in non-demented elderly subjects (Yassa et al., 2010a, 2011). While the spatial resolution of fMRI does not easily allow for the CA3 and dentate subregions to be isolated, it is likely that CA3 hyperactivity is the genesis of this increased BOLD signal. In fact, other research has shown reduced activity in the dentate gyrus with age (Small et al., 2004; Penner et al., 2010). While fMRI data have been foundational for linking aberrant CA3 activity to impairments in the ability to discriminate between similar stimuli, additional mechanistic studies in animal models are necessary to understand the cellular basis of the enhanced BOLD signal in aged CA3. The current data suggest that in addition to higher firing rates, the proportion of active neurons is increased in aged-impaired rats.

While the higher percentages of cells that where positive for the neural activity-dependent immediate-early gene *Arc* in A-poor performing rats is consistent with reports of CA3 hyperactivity, unlike previous studies (Wilson et al., 2005; Robitsek et al., 2015) the A-poor rats in the present experiments were able to update the active CA3 principal cell population when the environment changed. One way in which the present study differed from previous reports was that both environments were novel. Studies reporting that CA3 neurons do not “remap” between different environments have compared firing patterns between a familiar environment and a novel environment (Wilson et al., 2005; Robitsek et al., 2015). Repeated experience within the same environment may strengthen the association between the stimuli and neurons to an extent that favors generalization whereby the same ensemble is retrieved when presented with similar, yet distinct stimuli. It is conceivable that a single exposure is not sufficient to bias the network to retrieve a previous representation. These findings are consistent with data from humans showing that repetition of a target stimulus improved recognition but decreased the ability to discriminate the target from similar lures (Reagh and Yassa, 2014b). This observation suggests that a failure to remap would only be observed in aged animals when the first environment explored had been experienced multiple times prior to the introduction of the second novel environment.

Another interesting finding regarding the overlapping activity patterns between the two different environments was the decreased similarity across epochs in distal compared to proximal CA3. Several reports have documented distinct activity dynamics along the transverse axis of CA3 such that proximal CA3 is more likely to pattern separate than the more distal portions in situation with cue conflict or overlapping input patterns (Marrone et al., 2014; Lee et al., 2015). Importantly, proximal CA3 receives relatively more input from the dentate gyrus (Claiborne et al., 1986; Witter, 2007) and distal CA3 receives relatively more input from the LEC (Ishizuka et al., 1995; Witter, 2007). In the current experiment, the sensory input and all of the allocentric cues were distinct between the two epochs of behavior. This may have promoted distinct representations across epochs in entorhinal cortical neurons that project to CA3. This idea is in fact supported by the LEC data, which showed greater orthogonalization among projection neurons to CA3 compared to all cells in LEC (Figure 6).

While the aged LEC neurons showed distinct neural populations that were active between the two environments across all behavioral groups, the aged-poor rats had a higher percent of LEC neurons that were active during the first epoch, relative to the other groups (Figure 6). Because this was observed in the subset of LEC neurons that project directly to CA3, it suggests that enhanced afferent drive to CA3, along with interneuron dysfunction (Cadacio et al., 2003; Koh et al., 2010; Spiegel et al., 2013; Thome et al., 2015) and altered membrane currents (Simkin et al., 2015), contributes to aberrant excitation. Interestingly, in the aged-poor rats only, there was a significant reduction in the percent of active cells between epochs 1 and 2. Neurophysiological recordings from the medial temporal lobe have shown that entorhinal cortical neurons have higher firing rates for novel vs. familiar stimuli (Xiang and Brown, 1998). In this context of this previous work, the reduction in active cells between epochs 1 and 2 could reflect the aged-impaired rats falsely identifying the second novel environment as familiar. A similar effect has been shown for objects, in which aged rats behave as if novel objects are familiar (Burke et al., 2010, 2011). These data leave open the question as to whether the MEC also shows aberrant activity patterns in aged-poor performing rats. While available behavioral data suggest that the MEC may not be as vulnerable in old age (Reagh and Yassa, 2014a, 2017; Reagh et al., 2015), relative to the LEC, this question warrants additional investigation.

Finally, this study investigated the allocation of cell activity across environments. As described previously (Witharana et al., 2016), the conventional theory of how place fields are allocated are a uniform random draw with replacement model in which no cell has more or less of a chance at being active in an environment than any other cell. However, the preconfigured, log-normally distributed firing rates of neurons in the hippocampus and entorhinal cortex (Mizuseki and Buzsáki, 2013; Buzsáki and Mizuseki, 2014) directly challenge traditional theories of memory encoding (namely “orthogonality”; McNaughton, 1998) and uniform random draw with replacement. High-firing rate cells in one environment will remain high-firing rate in another. Contrary to prior results (Witharana et al., 2016), we failed to detect an under recruitment of CA3 neurons across environments for either proximal or distal subregions. Neurons were found to remap as expected by uniform random draw with replacement (Figure 5). When examining all neurons in the LEC, however, we found that—on the whole—neurons were active in a manner that consists with a preconfigured network architecture. Using CTB retrograde tracing, we were able to advance this analysis one-step further by examining the specific LEC neurons with projections into CA3. Incredibly, these neurons shifted their activity more than expected in either the traditional model or the preconfigured representation. As called for by Buzsáki and Mizuseki (2014), it is necessary to consider how prewired configurations support learning and memory. The present data are in agreement with this idea and emphasize the importance of this framework for understanding the etiological of cognitive aging.

## Author contributions

AM and SB designed the experiment, analyzed the data, and wrote the paper. SJ, AH, KL, JR, DC, KF, and JM assisted in collecting and analyzing data. BC provided surgical training and data analysis support.

### Conflict of interest statement

The authors declare that the research was conducted in the absence of any commercial or financial relationships that could be construed as a potential conflict of interest.

## References

[B1] Alme C. B. Buzzetti R. A. Marrone D. F. Leutgeb J. K. Chawla M. K. Schaner M. J.. (2010). Hippocampal granule cells opt for early retirement. Hippocampus 20, 1109–1123. 10.1002/hipo.20810 20872737

[B2] Alme C. B. Miao C. Jezek K. Treves A. Moser E. I. Moser M. B. (2014). Place cells in the hippocampus: eleven maps for eleven rooms. Proc. Natl. Acad. Sci. U.S.A. 111, 18428–18435. 10.1073/pnas.1421056111 25489089 PMC4284589

[B3] Bakker A. Krauss G. L. Albert M. S. Speck C. L. Jones L. R. Stark C. E.. (2012). Reduction of hippocampal hyperactivity improves cognition in amnestic mild cognitive impairment. Neuron 74, 467–474. 10.1016/j.neuron.2012.03.023 22578498 PMC3351697

[B4] Barnes C. A. (1979). Memory deficits associated with senescence: a neurophysiological and behavioral study in the rat. J. Comp. Physiol. Psychol. 93, 74–104. 10.1037/h0077579 221551

[B5] Barnes C. A. McNaughton B. L. (1980). Physiological compensation for loss of afferent synapses in rat hippocampal granule cells during senescence. J. Physiol. 309, 473–485. 10.1113/jphysiol.1980.sp013521 7252877 PMC1274597

[B6] Barnes C. A. Nadel L. Honig W. K. (1980). Spatial memory deficit in senescent rats. Can. J. Psychol. 34, 29–39. 10.1037/h0081022 7388694

[B7] Bartko S. J. Winters B. D. Cowell R. A. Saksida L. M. Bussey T. J. (2007a). Perceptual functions of perirhinal cortex in rats: zero-delay object recognition and simultaneous oddity discriminations. J. Neurosci. 27, 2548–2559. 10.1523/JNEUROSCI.5171-06.2007 17344392 PMC6672512

[B8] Bartko S. J. Winters B. D. Cowell R. A. Saksida L. M. Bussey T. J. (2007b). Perirhinal cortex resolves feature ambiguity in configural object recognition and perceptual oddity tasks. Learn. Mem. 14, 821–832. 10.1523/JNEUROSCI.5171-06.2007 18086825 PMC2151019

[B9] Behrmann M. Lee A. C. Geskin J. Z. Graham K. S. Barense M. D. (2016). Temporal lobe contribution to perceptual function: a tale of three patient groups. Neuropsychologia 90, 33–45. 10.1016/j.neuropsychologia.2016.05.002 27150707

[B10] Braak H. Alafuzoff I. Arzberger T. Kretzschmar H. Del Tredici K. (2006). Staging of Alzheimer disease-associated neurofibrillary pathology using paraffin sections and immunocytochemistry. Acta Neuropathol. 112, 389–404. 10.1007/s00401-006-0127-z 16906426 PMC3906709

[B11] Braak H. Braak E. Bohl J. (1993). Staging of Alzheimer-related cortical destruction. Eur. Neurol. 33, 403–408. 10.1159/000116984 8307060

[B12] Bunzeck N. Duzel E. (2006). Absolute coding of stimulus novelty in the human substantia nigra/VTA. Neuron 51, 369–379. 10.1016/j.neuron.2006.06.021 16880131

[B13] Burke S. N. Barnes C. A. (2006). Neural plasticity in the ageing brain. Nat. Rev. Neurosci. 7, 30–40. 10.1038/nrn1809 16371948

[B14] Burke S. N. Barnes C. A. (2010). Senescent synapses and hippocampal circuit dynamics. Trends Neurosci. 33, 153–161. 10.1016/j.tins.2009.12.003 20071039 PMC3076741

[B15] Burke S. N. Chawla M. K. Penner M. R. Crowell B. E. Worley P. F. Barnes C. A.. (2005). Differential encoding of behavior and spatial context in deep and superficial layers of the neocortex. Neuron 45, 667–674. 10.1016/j.neuron.2005.01.042 15748843

[B16] Burke S. N. Hartzell A. L. Lister J. P. Hoang L. T. Barnes C. A. (2012). Layer V perirhinal cortical ensemble activity during object exploration: a comparison between young and aged rats. Hippocampus 22, 2080–2093. 10.1002/hipo.22066 22987683 PMC3523702

[B17] Burke S. N. Wallace J. L. Hartzell A. L. Nematollahi S. Plange K. Barnes C. A. (2011). Age-associated deficits in pattern separation functions of the perirhinal cortex: a cross-species consensus. Behav. Neurosci. 125, 836–847. 10.1037/a0026238 22122147 PMC3255096

[B18] Burke S. N. Wallace J. L. Nematollahi S. Uprety A. R. Barnes C. A. (2010). Pattern separation deficits may contribute to age-associated recognition impairments. Behav. Neurosci. 124, 559–573. 10.1037/a0020893 20939657 PMC3071152

[B19] Bussey T. J. Saksida L. M. Murray E. A. (2006). Perirhinal cortex and feature-ambiguous discriminations. Learn. Mem. 13, 103–105; author reply: 106–107. 10.1101/lm.163606 16585785

[B20] Buzsaki G. (1984). Feed-forward inhibition in the hippocampal formation. Prog. Neurobiol. 22, 131–153. 10.1016/0301-0082(84)90023-6 6433403

[B21] Buzsaki G. Czeh G. (1981). Commissural and perforant path interactions in the rat hippocampus. Field potentials and unitary activity. Exp. Brain Res. 43, 429–438. 7262237 10.1007/BF00238387

[B22] Buzsaki G. Czopf J. Kondakor I. Kellenyi L. (1986). Laminar distribution of hippocampal rhythmic slow activity (RSA) in the behaving rat: current-source density analysis, effects of urethane and atropine. Brain Res. 365, 125–137. 10.1016/0006-8993(86)90729-8 3947979

[B23] Buzsáki G. Mizuseki K. (2014). The log-dynamic brain: how skewed distributions affect network operations. Nat. Rev. Neurosci. 15, 264–278. 10.1038/nrn3687 24569488 PMC4051294

[B24] Cadacio C. L. Milner T. A. Gallagher M. Pierce J. P. (2003). Hilar neuropeptide Y interneuron loss in the aged rat hippocampal formation. Exp. Neurol. 183, 147–158. 10.1016/S0014-4886(03)00126-2 12957498

[B25] Claiborne B. J. Amaral D. G. Cowan W. M. (1986). A light and electron microscopic analysis of the mossy fibers of the rat dentate gyrus. J. Comp. Neurol. 246, 435–458. 10.1002/cne.902460403 3700723

[B26] Cole A. J. Saffen D. W. Baraban J. M. Worley P. F. (1989). Rapid increase of an immediate early gene messenger RNA in hippocampal neurons by synaptic NMDA receptor activation. Nature 340, 474–476. 10.1038/340474a0 2547165

[B27] Conte W. L. Kamishina H. Reep R. L. (2009a). The efficacy of the fluorescent conjugates of cholera toxin subunit B for multiple retrograde tract tracing in the central nervous system. Brain Struct. Funct. 213, 367–373. 10.1007/s00429-009-0212-x 19621243

[B28] Conte W. L. Kamishina H. Reep R. L. (2009b). Multiple neuroanatomical tract-tracing using fluorescent Alexa Fluor conjugates of cholera toxin subunit B in rats. Nat. Protoc. 4, 1157–1166. 10.1007/s00429-009-0212-x 19617887

[B29] Dellu-Hagedorn F. Trunet S. Simon H. (2004). Impulsivity in youth predicts early age-related cognitive deficits in rats. Neurobiol. Aging 25, 525–537. 10.1016/j.neurobiolaging.2003.06.006 15013574

[B30] Devlin J. T. Price C. J. (2007). Perirhinal contributions to human visual perception. Curr. Biol. 17, 1484–1488. 10.1016/j.cub.2007.07.066 17764947 PMC1971135

[B31] Dieguez D. Jr. Barea-Rodriguez E. J. (2004). Aging impairs the late phase of long-term potentiation at the medial perforant path-CA3 synapse in awake rats. Synapse 52, 53–61. 10.1002/syn.20004 14755632 PMC1913478

[B32] Ennaceur A. Delacour J. (1988). A new one-trial test for neurobiological studies of memory in rats. 1: behavioral data. Behav. Brain Res. 31, 47–59. 10.1016/0166-4328(88)90157-X 3228475

[B33] Foster T. C. Barnes C. A. Rao G. McNaughton B. L. (1991). Increase in perforant path quantal size in aged F-344 rats. Neurobiol. Aging 12, 441–448. 10.1016/0197-4580(91)90071-Q 1770978

[B34] Gallagher M. Burwell R. Burchinal M. (1993). Severity of spatial learning impairment in aging: development of a learning index for performance in the Morris water maze. Behav. Neurosci. 107, 618–626. 10.1037/0735-7044.107.4.618 8397866

[B35] Graham K. S. Barense M. D. Lee A. C. (2010). Going beyond LTM in the MTL: A synthesis of neuropsychological and neuroimaging findings on the role of the medial temporal lobe in memory and perception. Neuropsychologia 48, 831–853. 10.1016/j.neuropsychologia.2010.01.001 20074580

[B36] Guzowski J. F. McNaughton B. L. Barnes C. A. Worley P. F. (1999). Environment-specific expression of the immediate-early gene Arc in hippocampal neuronal ensembles. Nat. Neurosci. 2, 1120–1124. 10.1038/16046 10570490

[B37] Guzowski J. F. McNaughton B. L. Barnes C. A. Worley P. F. (2001). Imaging neural activity with temporal and cellular resolution using FISH. Curr. Opin. Neurobiol. 11, 579–584. 10.1016/S0959-4388(00)00252-X 11595491

[B38] Hernandez A. R. Maurer A. P. Reasor J. E. Turner S. M. Barthle S. E. Johnson S. A.. (2015). Age-related impairments in object-place associations are not due to hippocampal dysfunction. Behav. Neurosci. 129, 599–610. 10.1037/bne0000093 26413723 PMC4945158

[B39] Hickman D. L. Swan M. (2010). Use of a body condition score technique to assess health status in a rat model of polycystic kidney disease. J. Am. Assoc. Lab. Anim. Sci. 49, 155–159. 20353688 PMC2846001

[B40] Ishizuka N. Cowan W. M. Amaral D. G. (1995). A quantitative analysis of the dendritic organization of pyramidal cells in the rat hippocampus. J. Comp. Neurol. 362, 17–45. 10.1002/cne.903620103 8576427

[B41] Johnson S. A. Turner S. M. Santacroce L. A. Carty K. N. Shafiq L. Bizon J. L.. (2017). Rodent age-related impairments in discriminating perceptually similar objects parallel those observed in humans. Hippocampus 27, 759–776. 10.1002/hipo.22729 28342259 PMC5479708

[B42] Kent B. A. Hvoslef-Eide M. Saksida L. M. Bussey T. J. (2016). The representational-hierarchical view of pattern separation: not just hippocampus, not just space, not just memory? Neurobiol. Learn. Mem. 129, 99–106. 10.1016/j.nlm.2016.01.006 26836403

[B43] Khan U. A. Liu L. Provenzano F. A. Berman D. E. Profaci C. P. Sloan R.. (2014). Molecular drivers and cortical spread of lateral entorhinal cortex dysfunction in preclinical Alzheimer's disease. Nat. Neurosci. 17, 304–311. 10.1038/nn.3606 24362760 PMC4044925

[B44] Koh M. T. Haberman R. P. Foti S. McCown T. J. Gallagher M. (2010). Treatment strategies targeting excess hippocampal activity benefit aged rats with cognitive impairment. Neuropsychopharmacology 35, 1016–1025. 10.1038/npp.2009.207 20032967 PMC2820138

[B45] Lee H. Wang C. Deshmukh S. S. Knierim J. J. (2015). Neural population evidence of functional heterogeneity along the CA3 transverse axis: pattern completion versus pattern separation. Neuron 87, 1093–1105. 10.1016/j.neuron.2015.07.012 26298276 PMC4548827

[B46] Lee I. Byeon J. S. (2014). Learning-dependent changes in the neuronal correlates of response inhibition in the prefrontal cortex and hippocampus. Exp. Neurobiol. 23, 178–189. 10.5607/en.2014.23.2.178 24963284 PMC4065833

[B47] Lee I. Kesner R. P. (2004). Encoding versus retrieval of spatial memory: double dissociation between the dentate gyrus and the perforant path inputs into CA3 in the dorsal hippocampus. Hippocampus 14, 66–76. 10.1002/hipo.10167 15058484

[B48] Leutgeb S. Leutgeb J. K. Treves A. Moser M. B. Moser E. I. (2004). Distinct ensemble codes in hippocampal areas CA3 and CA1. Science 305, 1295–1298. 10.1126/science.1100265 15272123

[B49] Lyford G. L. Yamagata K. Kaufmann W. E. Barnes C. A. Sanders L. K. Copeland N. G.. (1995). Arc, a growth factor and activity-regulated gene, encodes a novel cytoskeleton-associated protein that is enriched in neuronal dendrites. Neuron 14, 433–445. 10.1016/0896-6273(95)90299-6 7857651

[B50] Marrone D. F. Satvat E. Odintsova I. V. Gheidi A. (2014). Dissociation of spatial representations within hippocampal region CA3. Hippocampus 24, 1417–1420. 10.1002/hipo.22367 25220839

[B51] McClelland J. L. McNaughton B. L. O'Reilly R. C. (1995). Why there are complementary learning systems in the hippocampus and neocortex: insights from the successes and failures of connectionist models of learning and memory. Psychol. Rev. 102, 419–457. 10.1037/0033-295X.102.3.419 7624455

[B52] McNaughton B. L. (1998). The neurophysiology of reminiscence. Neurobiol. Learn. Mem. 70, 252–267. 10.1006/nlme.1998.3851 9753600

[B53] McNaughton B. L. Morris R. G. (1987). Hippocampal synaptic enhancement and information storage within a distributed memory system. Trends Neurosci. 10, 408–415. 10.1016/0166-2236(87)90011-7

[B54] McTighe S. M. Cowell R. A. Winters B. D. Bussey T. J. Saksida L. M. (2010). Paradoxical false memory for objects after brain damage. Science 330, 1408–1410. 10.1126/science.1194780 21127256

[B55] Mesina L. Wilber A. A. Clark B. J. Dube S. Demecha A. J. Stark C. E.. (2016). A methodological pipeline for serial-section imaging and tissue realignment for whole-brain functional and connectivity assessment. J. Neurosci. Methods 266, 151–160. 10.1016/j.jneumeth.2016.03.021 27039972 PMC5695690

[B56] Mizuseki K. Buzsáki G. (2013). Preconfigured, skewed distribution of firing rates in the hippocampus and entorhinal cortex. Cell Rep. 4, 1010–1021. 10.1016/j.celrep.2013.07.039 23994479 PMC3804159

[B57] Norman K. A. O'Reilly R. C. (2003). Modeling hippocampal and neocortical contributions to recognition memory: a complementary-learning-systems approach. Psychol. Rev. 110, 611–646. 10.1037/0033-295X.110.4.611 14599236

[B58] O'Reilly R. C. Norman K. A. (2002). Hippocampal and neocortical contributions to memory: advances in the complementary learning systems framework. Trends Cogn. Sci. 6, 505–510. 10.1016/S1364-6613(02)02005-3 12475710

[B59] Paxinos G. Watson C. (2007). The Rat Brain in Stereotaxic Coordinates. Burlington, MA: Elsevier Inc.

[B60] Penner M. R. Roth T. L. Chawla M. K. Hoang L. T. Roth E. D. Lubin F. D.. (2010). Age-related changes in Arc transcription and DNA methylation within the hippocampus. Neurobiol. Aging 32, 2198–2210. 10.1016/j.neurobiolaging.2010.01.009 20189687 PMC2888808

[B61] Rapp P. R. Deroche P. S. Mao Y. Burwell R. D. (2002). Neuron number in the parahippocampal region is preserved in aged rats with spatial learning deficits. Cereb. Cortex 12, 1171–1179. 10.1093/cercor/12.11.1171 12379605

[B62] Rapp P. R. Gallagher M. (1996). Preserved neuron number in the hippocampus of aged rats with spatial learning deficits. Proc. Natl. Acad. Sci. U.S.A. 93, 9926–9930. 10.1073/pnas.93.18.9926 8790433 PMC38531

[B63] Reagh Z. Yassa M. (2017). Selective vulnerabilities and biomarkers in neurocognitive aging. F1000Res 6, 491. 10.12688/f1000research.10652.1 28491288 PMC5399968

[B64] Reagh Z. M. Ho H. D. Leal S. L. Noche J. A. Chun A. Murray E. A.. (2015). Greater loss of object than spatial mnemonic discrimination in aged adults. Hippocampus 26, 417–422. 10.1002/hipo.22562 26691235 PMC5918289

[B65] Reagh Z. M. Yassa M. A. (2014a). Object and spatial mnemonic interference differentially engage lateral and medial entorhinal cortex in humans. Proc. Natl. Acad. Sci. U.S.A. 111, E4264–E4273. 10.1073/pnas.1411250111 25246569 PMC4210036

[B66] Reagh Z. M. Yassa M. A. (2014b). Repetition strengthens target recognition but impairs similar lure discrimination: evidence for trace competition. Learn. Mem. 21, 342–346. 10.1101/lm.034546.114 24934334 PMC4061427

[B67] Reid J. M. Jacklin D. L. Winters B. D. (2012). Crossmodal object recognition in rats with and without multimodal object pre-exposure: no effect of hippocampal lesions. Neurobiol. Learn. Mem. 98, 311–319. 10.1016/j.nlm.2012.09.001 22975081

[B68] Robitsek J. Ratner M. H. Stewart T. Eichenbaum H. Farb D. H. (2015). Combined administration of levetiracetam and valproic acid attenuates age-related hyperactivity of CA3 place cells, reduces place field area, and increases spatial information content in aged rat hippocampus. Hippocampus 25, 1541–1555. 10.1002/hipo.22474 25941121 PMC4633399

[B69] Rolls E. T. (2013). The mechanisms for pattern completion and pattern separation in the hippocampus. Front. Syst. Neurosci. 7:74. 10.3389/fnsys.2013.00074 24198767 PMC3812781

[B70] Rolls E. T. (2015). Pattern separation, completion, and categorisation in the hippocampus and neocortex. Neurobiol. Learn. Mem. 129, 4–28. 10.1016/j.nlm.2015.07.008 26190832

[B71] Rosenzweig E. S. Barnes C. A. (2003). Impact of aging on hippocampal function: plasticity, network dynamics, and cognition. Prog. Neurobiol. 69, 143–179. 10.1016/S0301-0082(02)00126-0 12758108

[B72] Simkin D. Hattori S. Ybarra N. Musial T. F. Buss E. W. Richter H.. (2015). Aging-related hyperexcitability in CA3 pyramidal neurons is mediated by enhanced A-type K^+^ channel function and expression. J. Neurosci. 35, 13206–13218. 10.1523/JNEUROSCI.0193-15.2015 26400949 PMC4579378

[B73] Small S. A. Chawla M. K. Buonocore M. Rapp P. R. Barnes C. A. (2004). Imaging correlates of brain function in monkeys and rats isolates a hippocampal subregion differentially vulnerable to aging. Proc. Natl. Acad. Sci. U.S.A. 101, 7181–7186. 10.1073/pnas.0400285101 15118105 PMC406486

[B74] Smith T. D. Adams M. M. Gallagher M. Morrison J. H. Rapp P. R. (2000). Circuit-specific alterations in hippocampal synaptophysin immunoreactivity predict spatial learning impairment in aged rats. J. Neurosci. 20, 6587–6593. 10964964 10.1523/JNEUROSCI.20-17-06587.2000 PMC6772954

[B75] Spiegel A. M. Koh M. T. Vogt N. M. Rapp P. R. Gallagher M. (2013). Hilar interneuron vulnerability distinguishes aged rats with memory impairment. J. Comp. Neurol. 521, 3508–3523. 10.1002/cne.23367 23749483 PMC4801143

[B76] Stark S. M. Yassa M. A. Lacy J. W. Stark C. E. (2013). A task to assess behavioral pattern separation (BPS) in humans: data from healthy aging and mild cognitive impairment. Neuropsychologia 51, 2442–2449. 10.1016/j.neuropsychologia.2012.12.014 23313292 PMC3675184

[B77] Stark S. M. Yassa M. A. Stark C. E. (2010). Individual differences in spatial pattern separation performance associated with healthy aging in humans. Learn. Mem. 17, 284–288. 10.1101/lm.1768110 20495062 PMC2884287

[B78] Thome A. Gray D. T. Erickson C. A. Lipa P. Barnes C. A. (2015). Memory impairment in aged primates is associated with region-specific network dysfunction. Mol. Psychiatry 21, 1257–1262. 10.1038/mp.2015.160 26503764 PMC4848213

[B79] Toner C. K. Pirogovsky E. Kirwan C. B. Gilbert P. E. (2009). Visual object pattern separation deficits in nondemented older adults. Learn. Mem. 16, 338–342. 10.1101/lm.1315109 19403797

[B80] Treves A. Rolls E. T. (1994). Computational analysis of the role of the hippocampus in memory. Hippocampus 4, 374–391. 10.1002/hipo.450040319 7842058

[B81] Ullman-Culleré M. H. Foltz C. J. (1999). Body condition scoring: a rapid and accurate method for assessing health status in mice. Lab. Anim. Sci. 49, 319–323. 10403450

[B82] Vazdarjanova A. Guzowski J. F. (2004). Differences in hippocampal neuronal population responses to modifications of an environmental context: evidence for distinct, yet complementary, functions of CA3 and CA1 ensembles. J. Neurosci. 24, 6489–6496. 10.1523/JNEUROSCI.0350-04.2004 15269259 PMC6729865

[B83] Wilson I. A. Gallagher M. Eichenbaum H. Tanila H. (2006). Neurocognitive aging: prior memories hinder new hippocampal encoding. Trends Neurosci. 29, 662–670. 10.1016/j.tins.2006.10.002 17046075 PMC2614702

[B84] Wilson I. A. Ikonen S. Gallagher M. Eichenbaum H. Tanila H. (2005). Age-associated alterations of hippocampal place cells are subregion specific. J. Neurosci. 25, 6877–6886. 10.1523/JNEUROSCI.1744-05.2005 16033897 PMC6725350

[B85] Winters B. D. Reid J. M. (2010). A distributed cortical representation underlies crossmodal object recognition in rats. J. Neurosci. 30, 6253–6261. 10.1523/JNEUROSCI.6073-09.2010 20445051 PMC6632708

[B86] Witharana W. K. Cardiff J. Chawla M. K. Xie J. Y. Alme C. B. Eckert M.. (2016). Nonuniform allocation of hippocampal neurons to place fields across all hippocampal subfields. Hippocampus 26, 1328–1344. 10.1002/hipo.22609 27273259 PMC8769666

[B87] Witter M. P. (2007). Intrinsic and extrinsic wiring of CA3: indications for connectional heterogeneity. Learn. Mem. 14, 705–713. 10.1101/lm.725207 18007015

[B88] Wu C. W. Vasalatiy O. Liu N. Wu H. Cheal S. Chen D. Y.. (2011). Development of a MR-visible compound for tracing neuroanatomical connections *in vivo*. Neuron 70, 229–243. 10.1016/j.neuron.2011.03.010 21521610 PMC3419536

[B89] Xiang J. Z. Brown M. W. (1998). Differential neuronal encoding of novelty, familiarity and recency in regions of the anterior temporal lobe. Neuropharmacology 37, 657–676. 10.1016/S0028-3908(98)00030-6 9705004

[B90] Yassa M. A. Lacy J. W. Stark S. M. Albert M. S. Gallagher M. Stark C. E. (2010a). Pattern separation deficits associated with increased hippocampal CA3 and dentate gyrus activity in nondemented older adults. Hippocampus 21, 968–979. 10.1002/hipo.20808 20865732 PMC3010452

[B91] Yassa M. A. Mattfeld A. T. Stark S. M. Stark C. E. (2011). Age-related memory deficits linked to circuit-specific disruptions in the hippocampus. Proc. Natl. Acad. Sci. U.S.A. 108, 8873–8878. 10.1073/pnas.1101567108 21555581 PMC3102362

[B92] Yassa M. A. Muftuler L. T. Stark C. E. (2010b). Ultrahigh-resolution microstructural diffusion tensor imaging reveals perforant path degradation in aged humans *in vivo*. Proc. Natl. Acad. Sci. U.S.A. 107, 12687–12691. 10.1073/pnas.1002113107 20616040 PMC2906542

[B93] Yassa M. A. Stark C. E. (2011). Pattern separation in the hippocampus. Trends Neurosci. 34, 515–525. 10.1016/j.tins.2011.06.006 21788086 PMC3183227

[B94] Yoder W. M. Gaynor L. S. Burke S. N. Setlow B. Smith D. W. Bizon J. L. (2017). Interaction between age and perceptual similarity in olfactory discrimination learning in F344 rats: relationships with spatial learning. Neurobiol. Aging 53, 122–137. 10.1016/j.neurobiolaging.2017.01.023 28259065 PMC5393344

